# Effectiveness of mRNA COVID-19 Vaccines as First Booster Doses in England: An Observational Study in OpenSAFELY-TPP

**DOI:** 10.1097/EDE.0000000000001747

**Published:** 2024-06-24

**Authors:** Elsie M. F. Horne, William J. Hulme, Edward P. K. Parker, Ruth H. Keogh, Elizabeth J. Williamson, Venexia M. Walker, Tom M. Palmer, Rachel Denholm, Rochelle Knight, Helen J. Curtis, Alex J. Walker, Colm D. Andrews, Amir Mehrkar, Jessica Morley, Brian MacKenna, Sebastian C. J. Bacon, Ben Goldacre, Miguel A. Hernán, Jonathan A. C. Sterne, and the OpenSAFELY collaborative

**Affiliations:** From the a Population Health Sciences, University of Bristol, Oakfield House, Oakfield Grove, Bristol, United Kingdom; b National Institute of Health and Care Research Bristol Biomedical Research Centre, Bristol, United Kingdom; c The Bennett Institute for Applied Data Science, Nuffield Department of Primary Care Health Sciences, University of Oxford, United Kingdom; d London School of Hygiene and Tropical Medicine, Keppel Street, London, United Kingdom; e MRC Integrative Epidemiology Unit, Bristol Medical School, University of Bristol, Bristol, United Kingdom; f Health Data Research UK South West, United Kingdom; g National Institute of Health and Care Research Applied Research Collaboration West, University Hospitals Bristol and Weston, United Kingdom; h CAUSALab, Harvard T.H. Chan School of Public Health, Boston, MA; i Departments of Epidemiology and Biostatistics, Harvard T.H. Chan School of Public Health, Boston, MA

**Keywords:** COVID-19, Electronic health records, Target trial emulation, Vaccine, Vaccine effectiveness

## Abstract

**Background:**

The UK delivered its first “booster” COVID-19 vaccine doses in September 2021, initially to individuals at high risk of severe disease, then to all adults. The BNT162b2 Pfizer-BioNTech vaccine was used initially, then also Moderna mRNA-1273.

**Methods:**

With the approval of the National Health Service England, we used routine clinical data to estimate the effectiveness of boosting with BNT162b2 or mRNA-1273 compared with no boosting in eligible adults who had received two primary course vaccine doses. We matched each booster recipient with an unboosted control on factors relating to booster priority status and prior COVID-19 immunization. We adjusted for additional factors in Cox models, estimating hazard ratios up to 182 days (6 months) following booster dose. We estimated hazard ratios overall and within the following periods: 1–14, 15–42, 43–69, 70–97, 98–126, 127–152, and 155–182 days. Outcomes included a positive SARS-CoV-2 test, COVID-19 hospitalization, COVID-19 death, non-COVID-19 death, and fracture.

**Results:**

We matched 8,198,643 booster recipients with unboosted controls. Adjusted hazard ratios over 6-month follow-up were: positive SARS-CoV-2 test 0.75 (0.74, 0.75); COVID-19 hospitalization 0.30 (0.29, 0.31); COVID-19 death 0.11 (0.10, 0.14); non-COVID-19 death 0.22 (0.21, 0.23); and fracture 0.77 (0.75, 0.78). Estimated effectiveness of booster vaccines against severe COVID-19-related outcomes peaked during the first 3 months following the booster dose. By 6 months, the cumulative incidence of positive SARS-CoV-2 test was higher in boosted than unboosted individuals.

**Conclusions:**

We estimate that COVID-19 booster vaccination, compared with no booster vaccination, provided substantial protection against COVID-19 hospitalization and COVID-19 death but only limited protection against positive SARS-CoV-2 test. Lower rates of fracture in boosted than unboosted individuals may suggest unmeasured confounding. Observational studies should report estimated vaccine effectiveness against nontarget and negative control outcomes.

Booster vaccination doses are likely to play a key role in the ongoing management of SARS-CoV-2. In mid-September 2021 the national COVID-19 vaccination program in England administered its first booster doses in adults who had already received their two-dose primary vaccination course.^1^ Eligibility was initially restricted to those at highest risk of severe disease, then progressively extended. Vaccine prioritization schedules were guided by recommendations from the Joint Committee for Vaccine and Immunization expert working group.^2^ By 15 December 2021 every adult was eligible.^3^ Booster doses were initially available no earlier than 6 months after dose two, but this was reduced to 3 months on 8 December 2021, following concerns over the emergence of the Omicron variant.^4,5^ Since these first booster doses, subsequent boosters were offered to certain groups in the spring and autumn of 2022.

Understanding the effectiveness of booster doses, how effectiveness wanes over time, and whether effectiveness differs between population subgroups will be crucial to the scheduling and targeting of future booster vaccinations. In this study, we analyzed population-scale linked electronic health records to emulate a target trial assessing the effectiveness of booster vaccination with BNT162b2 or mRNA-1273, compared with no booster vaccination, against various outcomes.^6,7^

## METHODS

### Data Sources and Definitions

OpenSAFELY-TPP includes detailed pseudonymized primary care data from practices using The Phoenix Partnership (TPP) SystmOne general practice software, which covers around 40% of the population in England. These primary care data are linked via the National Health Service (NHS) number with inpatient hospital spell records (Hospital Episode Statistics dataset), national SARS-CoV-2 testing records (Second Generation Surveillance System), and national death registry records. Vaccination history and health and social care worker status (recorded for vaccine recipients at the time of vaccination) are available in the general practice record directly via the National Immunization Management System.

### Study Design

This is an observational matched cohort study using a target trial approach to address potential design-related biases. We used a sequential approach, in which we emulated a sequence of trials starting on each day of the study period, which started on 16 September 2021 (the start of the booster rollout in England) and ended on 28 February 2022 (after which there were few booster doses administered in people with two doses).^8,9^

### Study Population

Eligible individuals were alive, aged 18 years or over, and registered with a practice using TPP software on the trial start date. We excluded individuals if: they had not completed a primary course of two doses of BNT162b2 or ChAdOx1 COVID-19 vaccines at least 75 days before the trial start date (with at least 17 days between their first and second doses); they had received any additional COVID-19 vaccine doses between their second dose and trial start date; they were known to be a care home resident or health care worker (groups prioritized for early COVID-19 vaccination); were on end-of-life care or medically housebound; their age, sex, English Index of Multiple Deprivation (IMD), ethnicity, geographical region were missing; they had evidence of SARS-CoV-2 infection in the 30 days before the trial start date; or they were in hospital on an unplanned admission at the trial start date.

### Treatment Strategies

The treated group comprised individuals who received a third COVID-19 vaccine dose of either BNT162b2 or mRNA-1273 on the trial start date (the “boosted” group). Individuals in the boosted group were matched to individuals who were eligible for but did not receive a third COVID-19 vaccine dose on or before the trial start date (the control “unboosted” group).

### Matching Strategy

We matched those eligible for the boosted group 1-1 with individuals randomly selected from those eligible for the unboosted group on the following variables defined on the trial start date (see eAppendix; http://links.lww.com/EDE/C135 for the calipers used for continuous variables and levels used to exactly match categorical variables): age; age band; clinical vulnerability; brand of primary vaccine course; date of second dose; geographical region of England; evidence of COVID-19 infection before study start date. Individuals who were not successfully matched were excluded. Individuals included in the unboosted group of a trial were not eligible to be included in the unboosted group of a trial starting on a subsequent day but were eligible for matching in the boosted group of a subsequent trial if they received a dose of BNT162b2 or mRNA-1273.

### Outcomes

We examined booster effectiveness against positive SARS-CoV-2 test (either polymerase chain reaction [PCR] or lateral flow test), COVID-19 hospital admission (any mention of COVID-19 as the reason for admission), and COVID-19 death (any mention on the death certificate). We included the following additional outcomes: non-COVID-19 death (no mention of COVID-19 on the death certificate), non-COVID-19 death with an International Classification of Diseases Tenth Revision (ICD10) code corresponding to cardiovascular disease on the death certificate, non-COVID-19 death with a cancer ICD10 code on the death certificate, and fracture. The rationale was that differences between vaccine groups in these additional outcomes could indicate unmeasured confounding so that they serve as “negative control” outcomes.^10^ In addition, between-group differences in non-COVID-19 deaths could also indicate misattribution of the cause of death or real effects of vaccination.

### Covariates

Adjusted regression models included the following potential confounding factors in addition to the matching variables: sex; ethnicity; English Index of Multiple Deprivation; body mass index (BMI); learning disability; serious mental illness; immunosuppressed; current pregnancy; number of comorbid conditions in different organ systems; interval between first and second doses; days since a positive SARS-CoV-2 test; number of SARS-CoV-2 tests reported; one or more influenza vaccines in the past three influenza seasons (see eAppendix; http://links.lww.com/EDE/C135 for covariate levels).

### Statistical Analysis

Eligible individuals were followed up from the date of booster vaccination in the boosted, and from the date of matching in the unboosted (i.e., a “time-since-treatment” timescale). Follow-up ended at the earliest of: 182 days after the trial start date; 31 March 2022 (for the outcome positive SARS-CoV-2 test); 1 July 2022 (all other outcomes); death; practice deregistration; and receipt of third dose by the matched control. Thus, follow-up for both the boosted and unboosted individuals in a matched pair was censored if and when the unboosted individual received a booster dose.^8^

We estimated Kaplan–Meier (KM) cumulative incidence curves by vaccination status and compared vaccine brands using 182-day (6-month) differences in cumulative incidence per 1000. Hazard ratios comparing boosted and unboosted individuals were estimated using Cox regression. We estimated hazard ratios both overall and within the following intervals after third dose: 1–14, 15–42, 43–70, 71–98, 99–126, 127–154, and 155–182 days. We adjusted estimates by including the covariates listed above in the Cox model and stratified by trial date (i.e., matching date), geographical region, and brand of primary vaccine course.

### Variant-specific Analysis

We investigated the effect of virus variants by adding a time-varying variable that took the following values: “Delta” for follow-up between 16 September and 30 November 2021, “Delta-Omicron-transition” between 1 and 31 December 2021, “Omicron” on or after 1 January 2022.^11^ This was added as a stratification variable in the Cox model, interacted with the vaccine group variable, to allow estimation of era-specific vaccine effectiveness.

### Subgroup Analyses

We repeated the statistical analysis in subgroups defined by primary course brand, prior COVID-19 infection, age group, and clinical vulnerability. Based on the findings for cancer-related non-COVID-19 death, we additionally included a subgroup with no evidence of cancer in the previous 5 years. Subgroup definitions are given in the eAppendix; http://links.lww.com/EDE/C135.

### Disclosure Control

To satisfy strict re-identification minimization requirements for statistical outputs from OpenSAFELY’s Trusted Research Environment, we rounded counts to the nearest three, nine, 15, and so on. We rounded plots of cumulative event counts and the Kaplan–Meier cumulative incidence estimates such that each increment is based on at least six events. Event rates, risk differences, and risk ratios were derived from these rounded estimates.

### Software, code, and reproducibility

Data management and analyses were conducted in Python version 3.8.10 and R version 4.0.2. All code is shared openly for review and re-use under MIT open license at https://github.com/opensafely/vaccine-effectiveness-3dose.

## RESULTS

### Study Population and Matching

**FIGURE 1.**
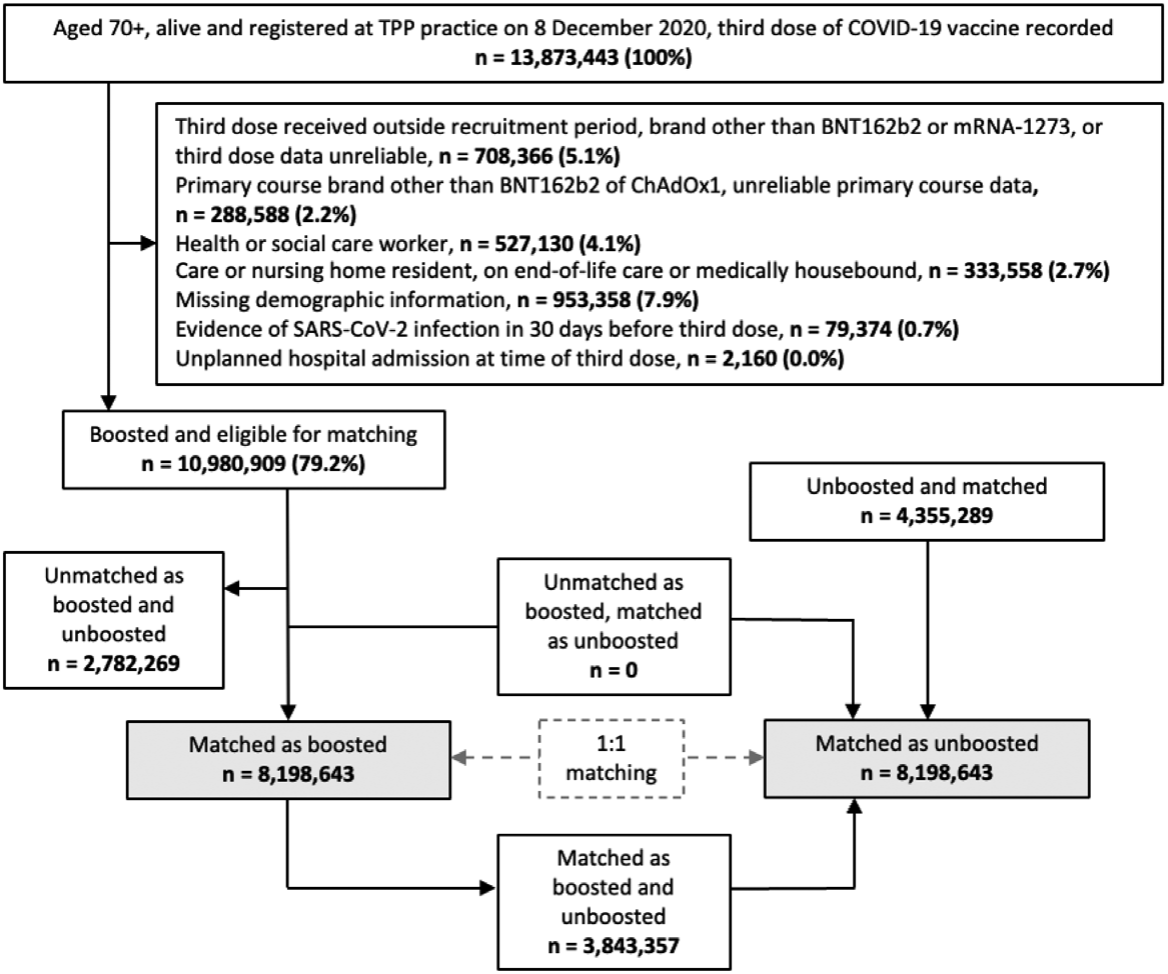
Flowchart showing selection of recipients of third dose of COVID-19 vaccination and matched controls.

**FIGURE 2.**
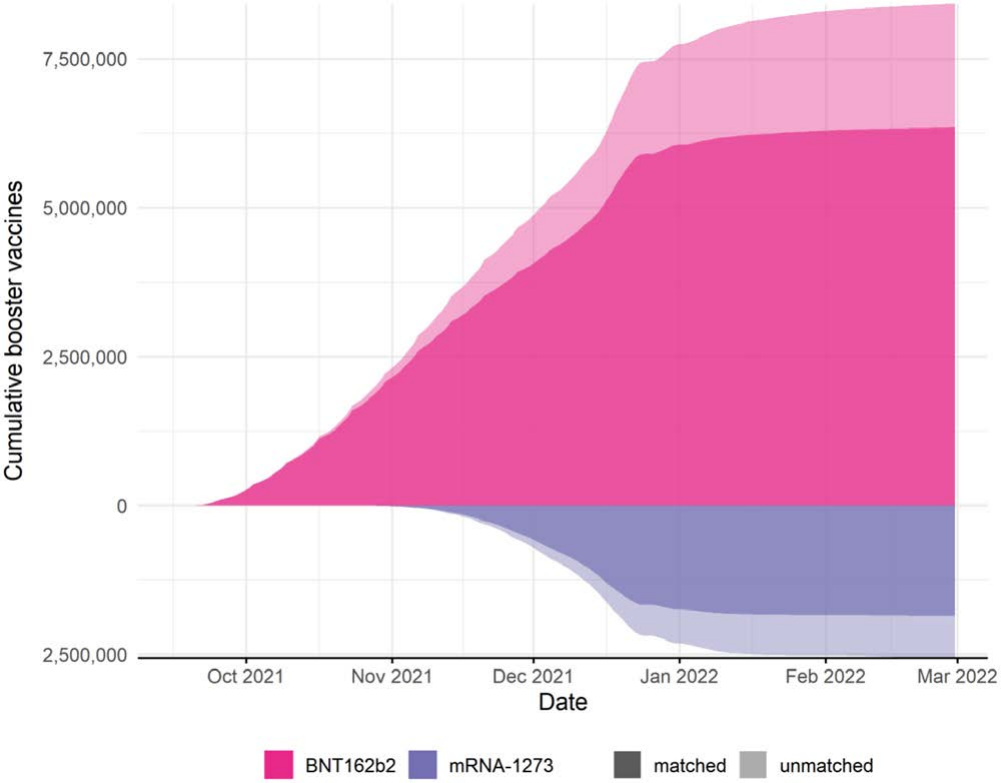
Cumulative number of recipients of third dose of COVID-19 vaccination eligible for inclusion, by vaccine type and matched status.

Of 13,873,443 adults registered at a TPP practice on 14 September 2021 with three recorded doses of COVID-19 vaccines, 10,980,909 (79.2%) were eligible for inclusion in the boosted group, and we matched 8,198,643 (59.1%) with unboosted controls. A total of 12,553,929 individuals were included in the study, 3,843,357 of whom contributed person-time to both the unboosted and boosted groups. Figure 1 shows the flow of individuals into the study, and Figure 2 shows the matching coverage, for both booster vaccine brands.

Matching factors were, by design, identically distributed in the boosted and unboosted groups at the start of follow-up (Table 1). The proportion of people with prior morbidities was generally similar between the groups. However, individuals in the unboosted group were less likely to be white (89.2% vs. 91.1%), were more deprived (17.9% vs. 14.1% in most deprived quintile), had higher rates of severe mental illness (1.0% vs. 0.8%), lower rates of immunosuppression (2.9% vs. 3.5%), higher rates of multimorbidity (10.3% vs. 9.8%), lower rates of prior influenza vaccination (48.4% vs. 54.9%), and lower rates of prior SARS-CoV-2 testing (21.3% vs. 22% had tested during the unvaccinated period). Of those with a documented prior SARS-CoV-2 infection, a longer time had elapsed for individuals in the unboosted group (8.4% vs. 7.8% had 91+ days since evidence of infection). At baseline, the median (interquartile range) days since the second dose was 182 (173, 191) in the unboosted group, and 188 (177, 196) in the boosted.

**TABLE 1.** Summary Statistics for Matching Variables and Model Covariates

Variable	Level	Unboosted	Boosted
N		8,198,643	8,198,643
Age (median, IQR)		55 (40, 68)	55 (40, 68)
Age band	Under 18	16,059 (0.2%)	16,059 (0.2%)
	18–39	1,990,647 (24.3%)	1,990,647 (24.3%)
	40–49	1,272,603 (15.5%)	1,272,603 (15.5%)
	50–54	832,395 (10.2%)	832,395 (10.2%)
	55–59	852,597 (10.4%)	852,597 (10.4%)
	60–64	765,093 (9.3%)	765,093 (9.3%)
	65–69	680,133 (8.3%)	680,133 (8.3%)
	70–74	687,459 (8.4%)	687,459 (8.4%)
	75–79	522,165 (6.4%)	522,165 (6.4%)
	80+	579,489 (7.1%)	579,489 (7.1%)
Sex	Female	4,230,195 (51.6%)	4,288,485 (52.3%)
	Male	3,968,445 (48.4%)	3,910,161 (47.7%)
Ethnicity	White	7,312,557 (89.2%)	7,472,427 (91.1%)
	Black	136,365 (1.7%)	93,345 (1.1%)
	South Asian	532,173 (6.5%)	431,787 (5.3%)
	Mixed	79,023 (1%)	70,683 (0.9%)
	Other	138,531 (1.7%)	130,395 (1.6%)
Deprivation	1 (most deprived)	1,465,563 (17.9%)	1,153,107 (14.1%)
	2	1,609,953 (19.6%)	1,455,663 (17.8%)
	3	1,818,993 (22.2%)	1,826,439 (22.3%)
	4	1,723,437 (21%)	1,879,707 (22.9%)
	5 (least deprived)	1,580,697 (19.3%)	1,883,733 (23%)
Region	North West	744,717 (9.1%)	744,717 (9.1%)
	Midlands	1,750,917 (21.4%)	1,750,917 (21.4%)
	North East and Yorkshire	1,523,565 (18.6%)	1,523,565 (18.6%)
	East of England	1,925,343 (23.5%)	1,925,343 (23.5%)
	London	455,025 (5.6%)	455,025 (5.6%)
	South East	543,465 (6.6%)	543,465 (6.6%)
	South West	1,255,611 (15.3%)	1,255,611 (15.3%)
Body mass index	Not obese	6,335,061 (77.3%)	6,338,193 (77.3%)
	Obese I (30–34.9)	1,110,861 (13.5%)	1,111,737 (13.6%)
	Obese II (35–39.9)	460,821 (5.6%)	453,855 (5.5%)
	Obese III (40+)	291,897 (3.6%)	294,855 (3.6%)
Learning disability		50,157 (0.6%)	45,531 (0.6%)
Severe mental illness		83,685 (1%)	67,299 (0.8%)
Immunosuppressed		234,279 (2.9%)	284,361 (3.5%)
Multimorbidity score	0	5,661,951 (69.1%)	5,676,765 (69.2%)
	1	1,693,629 (20.7%)	1,719,531 (21%)
	2+	843,057 (10.3%)	802,341 (9.8%)
Pregnancy		34,779 (0.4%)	33,879 (0.4%)
Clinically vulnerability status	Not clinically at-risk	5,552,901 (67.7%)	5,552,901 (67.7%)
	Clinically at-risk	1,940,679 (23.7%)	1,940,679 (23.7%)
	Clinically extremely vulnerable	705,069 (8.6%)	705,069 (8.6%)
Influenza vaccine		3,967,209 (48.4%)	4,498,005 (54.9%)
Third-dose vaccine type	BNT162b2	-	6,345,825 (77.4%)
	Moderna	-	1,852,821 (22.6%)
Primary course vaccine type	BNT162b2-BNT162b2	3,439,941 (42%)	3,439,941 (42%)
	ChAdOx1-ChAdOx1	4,758,699 (58%)	4,758,699 (58%)
Days between first and second dose		77 (66, 78)	76 (64, 78)
Days since second dose		182 (173, 191)	188 (177, 196)
Number of SARS-CoV-2 tests during unvaccinated period	0	6,452,385 (78.7%)	6,396,447 (78%)
	1	1,169,823 (14.3%)	1,204,791 (14.7%)
	2	303,915 (3.7%)	316,467 (3.9%)
	3+	272,523 (3.3%)	280,935 (3.4%)
Prior documented SARS-CoV-2 infection		918,135 (11.2%)	918,135 (11.2%)
Time since last evidence of SARS-CoV-2 infection	Never	7,312,647 (89.2%)	7,310,097 (89.2%)
	31–90 days	201,213 (2.5%)	248,121 (3%)
	91+ days	684,777 (8.4%)	640,419 (7.8%)

IQR indicates interquartile range.

### Estimated Booster Effectiveness

**FIGURE 3.**
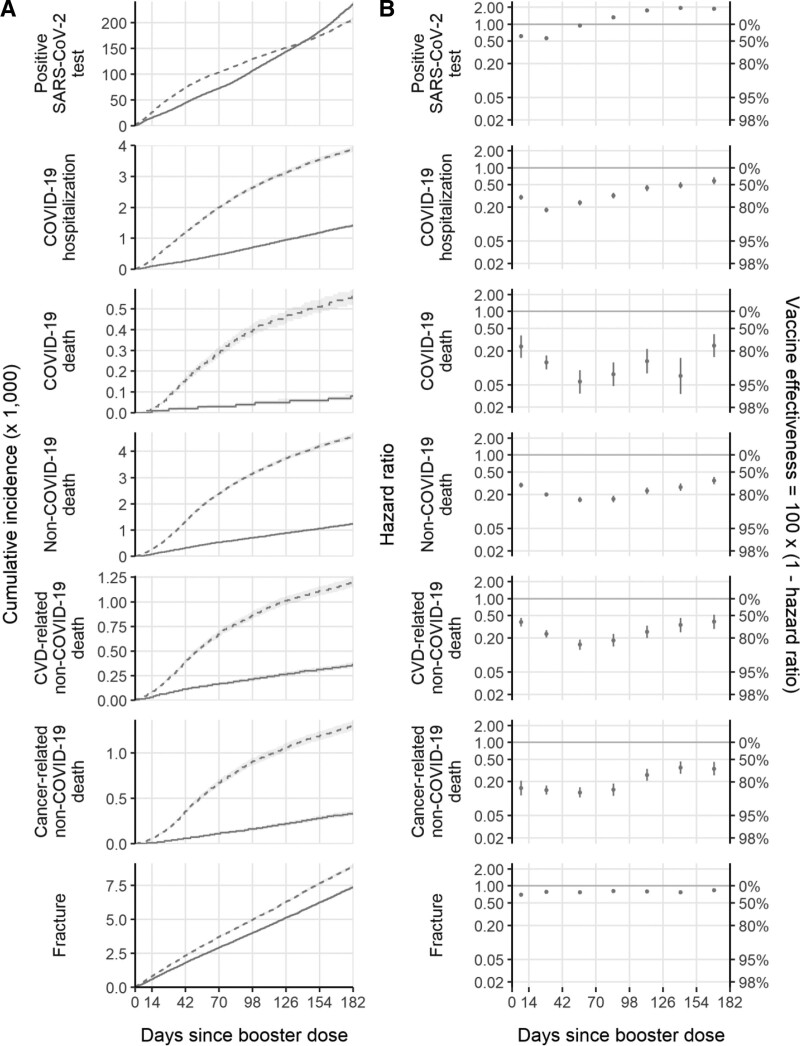
Cumulative incidence (A) and hazard ratios (B) of studied outcomes, comparing boosted and unboosted groups.

Follow-up was 1,402,445 person-years for the positive SAS-CoV-2 test (censored on 31 March 2022) and ranged from 2,104,847 to 2,111,190 person-years for the other outcomes (censored on 30 September 2022). There were 683,292 positive SARS-CoV-2 tests, 11,460 COVID-19 hospitalizations, 1,290 COVID-19 deaths, 12,378 non-COVID-19 deaths, and 35,424 fractures (Table 2). Differences in the cumulative incidence between the vaccine groups were apparent during the first few days of follow-up (Figure 3A).

**TABLE 2.** Event Counts, Estimated 6-month Cumulative Incidence Per 1000 (Kaplan–Meier) and 6-month Adjusted Hazard Ratios (Adjusted Cox)

	Positive SARS-CoV-2 Test	COVID-19 Hospitalization	COVID-19 Death	Non-COVID-19 Death	CVD-related Non-COVID-19 Death	Cancer-related Non-COVID-19 Death	Fracture
Event count in the unboosted	387,777	8,637	1,125	9,753	2,643	2,643	19,599
Event count in the boosted	295,515	2,823	165	2,625	807	621	15,825
Person-time in the unboosted (years)	683,706	1,050,453	1,052,175	1,052,175	1,052,175	1,052,175	1,048,575
Person-time in the boosted (years)	718,739	1,058,558	1,059,015	1,059,015	1,059,015	1,059,015	1,056,272
Cumulative incidence^a^ in the unboosted	207 (205, 210)	3.88 (3.79, 3.97)	0.56 (0.52, 0.59)	4.57 (4.47, 4.67)	1.20 (1.15, 1.25)	1.30 (1.24, 1.35)	8.92 (8.78, 9.05)
Cumulative incidence^a^ in the boosted	235 (233, 238)	1.41 (1.35, 1.46)	0.08 (0.07, 0.09)	1.24 (1.19, 1.30)	0.36 (0.33, 0.39)	0.33 (0.31, 0.36)	7.37 (7.25, 7.50)
Difference in cumulative incidence (boosted–unboosted)	28.3 (24.5, 32.1)	−2.48 (−2.58, −2.37)	−0.48 (−0.52, −0.44)	−3.33 (−3.44, −3.22)	−0.84 (−0.90, −0.79)	−0.96 (−1.02, −0.90)	−1.54 (−1.73, −1.36)
Unadjusted HR (boosted vs. unboosted)	0.73 (0.72, 0.73)	0.32 (0.31, 0.34)	0.14 (0.12, 0.17)	0.27 (0.26, 0.28)	0.30 (0.28, 0.33)	0.23 (0.21, 0.26)	0.80 (0.79, 0.82)
aHR (boosted vs. unboosted)	0.75 (0.74, 0.75)	0.30 (0.29, 0.31)	0.11 (0.10, 0.14)	0.22 (0.21, 0.23)	0.25 (0.23, 0.27)	0.18 (0.17, 0.20)	0.77 (0.75, 0.78)
aHR (31–90 days since prior infection vs. no prior infection)	0.28 (0.28, 0.29)	0.41 (0.35, 0.48)	0.40 (0.23, 0.69)	0.49 (0.42, 0.58)	0.68 (0.52, 0.89)	0.54 (0.41, 0.72)	0.99 (0.93, 1.05)
aHR (91+ days since prior infection vs. no prior infection)	0.66 (0.65, 0.66)	0.48 (0.44, 0.52)	0.25 (0.17, 0.36)	0.54 (0.50, 0.59)	0.61 (0.52, 0.71)	0.48 (0.41, 0.57)	1.07 (1.03, 1.11)

aHR indicates adjusted hazard ratio; CVD, cardiovascular disease; HR, hazard ratio

a 6-month cumulative incidence per 1000.

At 6 months, there were 28.3 (95% CI = 24.5, 32.1) more positive SARS-CoV-2 tests per 1000 in the boosted group than the unboosted. However, there were fewer events in the boosted group than the unboosted group for all severe outcomes (COVID-19 hospitalization −2.48 [−2.58, 2.37]; COVID-19 death −0.48 [−0.52, −0.44]) and control outcomes (non-COVID-19 death −3.33 [−3.44, −3.22]; fracture −1.54 [−1.73, −1.36]) (Table 2). The estimated 6-month aHRs (boosted vs. unboosted) were 0.75 (0.74, 0.75) for positive SARS-CoV-2 tests; 0.30 (0.29, 0.31) for COVID-19 hospitalization; 0.11 (0.10, 0.14) for COVID-19 death; 0.22 (0.21, 0.23) for non-COVID-19 death; 0.25 (0.23, 0.27) for CVD-related non-COVID-19 death, 0.18 (0.17, 0.20) for cancer-related non-COVID-19 death and 0.77 (0.75, 0.78) for fracture (Table 2). Unadjusted and adjusted hazard ratios were similar (Table 2).

We estimated adjusted hazard ratios comparing individuals with evidence of a prior infection 31–90 or 91+ days before the trial start date to those with no evidence of prior infection (Table 2; individuals with evidence of a prior infection within 30 days of the trial start date were excluded). The adjusted hazard ratios were: positive SARS-CoV-2 test 0.28 (0.28, 0.29) for 31–90 days versus 0.66 (0.65, 0.66) for 91+ days; COVID-19 hospitalization 0.41 (0.35, 0.48) for 31–90 days versus 0.48 (0.44, 0.52) for 91+ days; COVID-19 death 0.40 (0.23, 0.69) for 31–90 days versus 0.25 (0.17, 0.36) for 91+; non-COVID-19 death 0.49 (0.42, 0.58) for 31–90 days versus 0.54 (0.50, 0.59) for 91+; and fracture 0.99 (0.93, 1.05) for 31–90 days versus 1.07 (1.03, 1.11) for 91+.

Estimated adjusted hazard ratios comparing boosted with unboosted individuals were lowest during days 15–42 after booster dose for positive SARS-CoV-2 test (0.57 [95% CI = 0.56, 0.57]) and COVID-19 hospitalization (0.18 [0.16, 0.20]) and waned to 1.89 (1.76, 2.03) for positive test and 0.59 (0.51, 0.68) for COVID-19 hospitalization during days 155–182 after booster dose (Table 3). Estimated adjusted hazard ratios for COVID-19 death (0.06 [0.04, 0.09]) and non-COVID-19 death (0.16 [0.14, 0.18]) were lowest during days 43–70 after booster dose and waned to 0.25 (0.15, 0.39) for COVID-19 death and 0.35 (0.30, 0.41) for non-COVID-19 death by days 155–182 after booster dose. Estimated adjusted hazard ratios for fracture varied between 0.70 (0.67, 0.73) and 0.83 (0.78, 0.89) over 6 months since the booster dose. Unadjusted and adjusted hazard ratios were similar (Table 3).

**TABLE 3.** Period-specific Hazard Ratios (HR; Cox model)

Days Since Booster	Positive SARS-CoV-2 test	COVID-19 Hospitalization	COVID-19 Death	Non-COVID-19 Death	CVD-related Non-COVID-19 Death	Cancer-related Non-COVID-19 Death	Fracture
Unadjusted HR							
1–14	0.60 (0.59, 0.60)	0.31 (0.28, 0.34)	0.26 (0.16, 0.41)	0.32 (0.28, 0.35)	0.41 (0.34, 0.48)	0.17 (0.13, 0.23)	0.71 (0.68, 0.74)
15–42	0.55 (0.55, 0.56)	0.19 (0.17, 0.21)	0.14 (0.11, 0.19)	0.23 (0.21, 0.25)	0.27 (0.23, 0.31)	0.17 (0.14, 0.20)	0.81 (0.77, 0.84)
43–70	0.92 (0.91, 0.93)	0.26 (0.23, 0.29)	0.07 (0.04, 0.12)	0.20 (0.18, 0.22)	0.19 (0.15, 0.23)	0.17 (0.14, 0.21)	0.81 (0.77, 0.86)
71–98	1.27 (1.26, 1.29)	0.36 (0.32, 0.40)	0.10 (0.06, 0.17)	0.22 (0.19, 0.25)	0.24 (0.19, 0.31)	0.20 (0.15, 0.25)	0.86 (0.81, 0.92)
99–126	1.61 (1.57, 1.65)	0.49 (0.44, 0.56)	0.18 (0.11, 0.30)	0.31 (0.27, 0.36)	0.35 (0.27, 0.45)	0.37 (0.29, 0.47)	0.85 (0.79, 0.90)
127–154	1.71 (1.64, 1.78)	0.55 (0.49, 0.63)	0.10 (0.05, 0.21)	0.37 (0.32, 0.42)	0.46 (0.35, 0.62)	0.50 (0.39, 0.65)	0.82 (0.77, 0.88)
155–182	1.65 (1.53, 1.77)	0.66 (0.58, 0.77)	0.35 (0.22, 0.56)	0.48 (0.42, 0.56)	0.53 (0.40, 0.71)	0.49 (0.37, 0.64)	0.89 (0.83, 0.95)
Adjusted HR							
1–14	0.61 (0.61, 0.62)	0.30 (0.27, 0.33)	0.24 (0.15, 0.37)	0.29 (0.26, 0.32)	0.38 (0.32, 0.45)	0.15 (0.11, 0.21)	0.70 (0.67, 0.73)
15–42	0.57 (0.56, 0.57)	0.18 (0.16, 0.20)	0.12 (0.09, 0.17)	0.20 (0.18, 0.22)	0.24 (0.21, 0.28)	0.14 (0.12, 0.17)	0.78 (0.74, 0.81)
43–70	0.94 (0.93, 0.95)	0.24 (0.21, 0.27)	0.06 (0.04, 0.09)	0.16 (0.14, 0.18)	0.15 (0.12, 0.19)	0.13 (0.10, 0.16)	0.77 (0.73, 0.82)
71–98	1.32 (1.30, 1.34)	0.32 (0.29, 0.36)	0.08 (0.05, 0.13)	0.17 (0.15, 0.19)	0.18 (0.14, 0.24)	0.14 (0.11, 0.18)	0.81 (0.76, 0.87)
99–126	1.74 (1.70, 1.78)	0.44 (0.39, 0.50)	0.13 (0.08, 0.22)	0.23 (0.20, 0.26)	0.26 (0.20, 0.33)	0.26 (0.21, 0.34)	0.80 (0.75, 0.85)
127–154	1.94 (1.86, 2.02)	0.49 (0.43, 0.56)	0.07 (0.03, 0.15)	0.27 (0.23, 0.31)	0.34 (0.25, 0.46)	0.35 (0.28, 0.45)	0.77 (0.72, 0.83)
155–182	1.89 (1.76, 2.03)	0.59 (0.51, 0.68)	0.25 (0.15, 0.39)	0.35 (0.30, 0.41)	0.39 (0.29, 0.52)	0.34 (0.26, 0.45)	0.83 (0.78, 0.89)

CVD indicates cardiovascular disease; HR, hazard ratio.

Estimated effectiveness of booster doses against both SARS-CoV-2 infection and COVID-19 hospitalization was lower during the Omicron era compared with the Delta era (eTable 1; http://links.lww.com/EDE/C135 and eFigure 1; http://links.lww.com/EDE/C135). Estimated effectiveness increased as the age of the subgroups increased (eTable 2; http://links.lww.com/EDE/C135 and eFigure 2; http://links.lww.com/EDE/C135). Estimated effectiveness against positive SARS-CoV-2 test was greater in those with evidence of prior infection compared to those without but was lower against COVID-19 hospitalization (eTable 3; http://links.lww.com/EDE/C135 and eFigure 3; http://links.lww.com/EDE/C135). Estimated effectiveness against positive SARS-CoV-2 test increased with increasing clinical vulnerability (eTable 4; http://links.lww.com/EDE/C135 and eFigure 4; http://links.lww.com/EDE/C135). There were no clear differences in estimated effectiveness stratified by primary course brand (eTable 5; http://links.lww.com/EDE/C135 and eFigure 5; http://links.lww.com/EDE/C135). The cumulative incidence of cancer-related non-COVID-19 death was lower in the subgroup with no evidence of cancer in the past 5 years compared with the full cohort (eFigure 6; http://links.lww.com/EDE/C135). However, the estimated adjusted hazard ratios for cancer-related non-COVID-19 deaths were very similar in that subgroup compared to those estimated in the main analysis (eTable 6; http://links.lww.com/EDE/C135).

## DISCUSSION

In this observational cohort study of over 12 million adults, the first COVID-19 booster vaccination, compared with no booster vaccination, provided substantial protection, which peaked 3 months after booster vaccination, against COVID-19 hospitalization and COVID-19 death. Rates of non-COVID-19 deaths were substantially lower in boosted than unboosted individuals. However, booster vaccination offered only limited protection against positive SARS-CoV-2 tests. Lower rates of fracture in boosted than unboosted individuals may suggest some unmeasured confounding.

Previous studies estimated higher vaccine effectiveness (VE) (100 × [1-aHR]) against SARS-CoV-2 infection than this study. A phase III trial estimated VE for three versus two BNT162b2 doses over a median 2.5 months follow-up as 94.6% (95% CI = 88.5, 97.9). Andrews et al.^12^ used a “test-negative” observational study design to estimate booster VE against symptomatic COVID-19 infection 14–34 days after a BNT162b2 booster in those aged 18–49 years. They estimated VEs of 83% (82, 84) and 90% (89, 90) following the primary courses of BNT162b2 and ChAdOx1, respectively.^12^ Another test-negative study reported similar estimates of booster VE against symptomatic infection with the Delta variant (83% [81, 84] 16–49 years) ≥14 days after BNT162b2 or mRNA-1273, but lower estimates against the Omicron variant (56% [51,60] 16–49 years).^13^ Estimated booster VE against test positivity in this study was substantially lower at 43% (44,45) in those aged 18–49 years 14–42 days after a booster dose (combining asymptomatic and symptomatic testing from documented PCR and lateral flow tests), compared with test-negative designs in UK data. The test-negative design may be less susceptible to bias from unmeasured health-related behaviors than our study but may be subject to “collider” (selection) bias: for example, health-related behaviors are associated with COVID-19 testing, booster vaccination, and infection with SARS-CoV-2, and so testing is a collider (influenced by both boosting and by infection).^14^

Despite waning, booster doses remained effective at preventing COVID-19 hospitalization and COVID-19 death 6 months after receipt of the booster dose. The rate of non-COVID-19 death was also substantially lower in boosted than unboosted individuals during the 6 months of follow-up, with the lowest aHR 43–70 days after the booster dose. A previous study found reduced rates of non-COVID-19 death in individuals vaccinated with two doses compared with unvaccinated individuals.^15^ To our knowledge, other studies of booster effectiveness did not include non-COVID-19 death as an outcome.^6,7,12,13,16,17^

The U-shape pattern in period-specific aHRs was near-identical for all and CVD-related non-COVID-19 deaths (28% of non-COVID-19 deaths were CVD-related). In contrast, aHRs for cancer-related non-COVID-19 death were low in the first four periods (days 1–14, 15–42, 43–70, and 71–98), ranging between 0.13 (0.10, 0.16) and 0.15 (0.11, 0.21), then increased to 0.34 (0.26, 0.45) by days 155–182 after the booster dose. These results suggest unmeasured confounding, plausibly because individuals with advanced cancer are less likely to receive a booster dose than individuals without. This reasoning extends to other non-COVID-19 deaths, as individuals at high risk of death in the next 6 months may be less likely to receive a booster dose. Another study found elevated rates of non-COVID-19 death in people who had received their second dose more than 6 months before compared with unvaccinated individuals, likely for the same reason.^18^

Previous studies identified an elevated risk of various health complications following the SARS-CoV-2 infection.^19,20^ It is therefore possible that some non-COVID-19 deaths are related to complications of COVID-19. If the estimated protective effect of booster vaccination against non-COVID-19 outcomes were solely due to unmeasured confounding by differences in health status between boosted and unboosted individuals, we would expect to see larger effects of booster vaccination on fracture than were estimated here. Further investigation into the relationship between SARS-CoV-2 and subsequent health outcomes may help to explain the apparent protection of vaccination against non-COVID-19 deaths.

This study applied the “sequential” target trial approach to estimate booster VE, includes data from over 12 million individuals, and accounts for confounding through both an extensive matching framework and model adjustment. We conducted numerous subgroup analyses to investigate treatment effect modifications. A limitation is the incomplete recording of SARS-CoV-2 infection in linked electronic health records. Lateral flow and PCR tests were freely available in England during the study period, and individuals were encouraged to report the results of lateral flow tests and seek a confirmatory PCR test when these were positive. However, many asymptomatic infections and some symptomatic infections will not have been recorded. A previous study found that vaccinated individuals were twice as likely to report positive SARS-CoV-2 testing intentions compared with unvaccinated individuals.^21^ It is possible that this differential testing behavior extends to boosted and unboosted individuals. Further, SARS-CoV-2 testing was not widely available early in the pandemic, so prior infection is likely to be under-ascertained.

People with symptomatic but undiagnosed SARS-CoV-2 infection may have deferred booster vaccination. However, as this was not recorded, subsequent events related to such infections would have been counted in the unboosted group. This may partially explain the effectiveness that we estimated during days 1–14. However, BNT162b2 increases protection against COVID-19 within 7 days of the third dose, so the estimated effectiveness during days 1–14 may be due, at least in part, to the rapid effectiveness of booster vaccination.^22^

We could not fully adjust for smoking and other lifestyle factors that are associated with increased mortality. However, it is unclear whether these risk factors would confound the estimates of effectiveness against COVID-19-related outcomes. We excluded certain groups, such as health care workers and care home residents, in which testing behaviors, vaccination uptake, and infection risk were unusual or had substantial within-group heterogeneity that could not be adequately measured and controlled for. The generalizability of our results to these excluded groups is unclear. Due to small numbers, we did not study booster effectiveness in those who had received the mRNA-1273 vaccine as their primary course, nor in those who had received a heterologous primary course.

We censored matched individuals in the unboosted group if they became boosted, as well as the corresponding boosted individual. This ensured that the boosted group did not have substantially longer follow-up on average, and during different calendar periods, than the unboosted group. This censoring may be informative if postbaseline factors influence the uptake of boosting in the unboosted group,^8^ however, we did not attempt to mitigate any biases this induced. To conclude, the effectiveness of booster vaccines against severe COVID-19-related outcomes peaked during the first 3 months following the booster dose. We estimate that booster vaccines offered limited protection against the SARS-CoV-2 infection. Observational studies of VE should routinely report estimated effectiveness against nontarget and negative control outcomes, to clarify the potential for unmeasured confounding and identify possible nonspecific benefits of vaccination.

## ACKNOWLEDGMENTS

We are very grateful for all the support received from the TPP Technical Operations team throughout this work, and for generous assistance from the information governance and database teams at NHS England and the NHS England Transformation Directorate.

## Supplementary Material


