# Supplementary material for: Effectiveness of mRNA COVID-19 Vaccines as First Booster Doses in England: An Observational Study in OpenSAFELY-TPP
Source: Epidemiology. 2024 Jun 24;35(4):568–78. doi: 10.1097/EDE.0000000000001747 (PMC11191555; doi:10.1097/EDE.0000000000001747)
Supplement: Supplementary file 1 [file ede-35-568-s001.docx]

## eAppendix

[Methods 1](#_Toc162281610)

[Matching 1](#_Toc162281611)

[Covariates 2](#_Toc162281612)

[Timing of outcome events 2](#_Toc162281613)

[Censoring on fourth dose 2](#_Toc162281614)

[Disclosure control 2](#_Toc162281615)

[Subgroup analyses 3](#_Toc162281616)

[Information governance and ethical approval 3](#_Toc162281617)

[Results 4](#_Toc162281618)

[Variant-specific analysis 4](#_Toc162281619)

[Age subgroups 4](#_Toc162281620)

[Prior SARS-CoV-2 infection subgroups 5](#_Toc162281621)

[Clinical vulnerability subgroups 5](#_Toc162281622)

[Primary course subgroups 5](#_Toc162281623)

[No evidence of cancer in the 5 years prior to the trial start date 6](#_Toc162281624)

[References 19](#_Toc162281625)

### Methods

#### Matching

The continuous matching variables listed in the main text were matched using the following calipers:

- age (within 3 years);
- date of second dose (within 7 days);

The categorical matching variables listed in the main text were matched exactly using the following levels:

- JCVI age band (18-39, 40-49, 50-54, 55-59, 60-64, 65-69, 70-74, 75-79, 80+ years);
- clinical vulnerability (not clinically vulnerable, clinically vulnerable, clinically extremely vulnerable);
- brand of primary vaccine course (BNT162b2 or ChAdOx1);
- geographical region of England (North East and Yorkshire, Midlands, East, London, South East, South West, North West);
- evidence of COVID-19 infection prior to study start date (Yes, No)

#### Covariates

The covariates listed in the main text have the following levels:

- sex (Male, Female);
- ethnicity (White, Black, South Asian, Mixed, Other);
- English Index of Multiple Deprivation (IMD; 1 is most deprived, 2, 3, 4, 5 is most deprived);
- body mass index (BMI; Missing or not obese, 30-34.9, 35-39.9, 40+);
- learning disability (Yes, No);
- serious mental illness (Yes, No);
- immunosuppressed (Yes, No);
- current pregnancy (Yes, No);
- number of comorbid conditions in different organ systems (0, 1, 2+);
- interval between first and second doses;
- days since a positive SARS-CoV-2 test (no positive test, 31-90, >90 days since positive test);
- number of SARS-CoV-2 tests reported between 18 May 2020 and 5 December (0, 1, 2, 3+);
- one or more flu vaccines in the seasons 2018-2019, 2019-2020 or 2020-2021 (Yes, No)

#### Timing of outcome events

Outcome events occurring on the trial start date (the day of booster dose in the boosted group) were considered to have occurred after vaccination (i.e., vaccination was assumed to have occurred at the start of the day). This is necessarily true for death and likely true for hospitalisation outcomes. For positive SARS-CoV-2 tests the order of events is less clear, but the assumption is reasonable given the policy of a recent positive test precluding vaccination.

#### Censoring on fourth dose

We did not censor individuals in the boosted group when they received a fourth vaccine dose. The rationale is that during the follow-up period, only the individuals who were most vulnerable to severe COVID-19 were eligible for fourth dose, and therefore censoring would have been informative.

#### Disclosure control

Any reported figures based on counts below 6 were redacted or rounded for disclosure control. Such rounding reduces secondary disclosure risk (e.g., by using two unredacted values to derive a redacted value). Kaplan-Meier survival estimates were rounded upwards by deferring event-times until there were at least 6 events, so that each KM step is based on at least 6 events. All other quantities were derived from these rounded KM estimates.

#### Subgroup analyses

The subgroups were as follows:

- primary course brand (ChAdOx1, BNT162b2; this subgroup analysis was restricted to JCVI groups 3-6, as JCVI groups 7+ received either predominantly BNT162b2or predominantly ChAdOx1);
- evidence of prior COVID-19 infection (yes or no);
- age group (18-49, 50-64, 65-79 and 80+ years);
- clinical vulnerability (not clinically vulnerable, at-risk, clinically extremely vulnerable)

#### Information governance and ethical approval

This study was approved by the Health Research Authority (REC reference 22/PR/0095) and by the University of Bristol's Faculty of Health Sciences Ethics Committee (reference 117269).

NHS England is the data controller for OpenSAFELY-TPP; TPP is the data processor; all study authors using OpenSAFELY have the approval of NHS England. This implementation of OpenSAFELY is hosted within the TPP environment which is accredited to the ISO 27001 information security standard and is NHS IG Toolkit compliant;^1^

Patient data has been pseudonymised for analysis and linkage using industry standard cryptographic hashing techniques; all pseudonymised datasets transmitted for linkage onto OpenSAFELY are encrypted; access to the platform is via a virtual private network (VPN) connection, restricted to a small group of researchers; the researchers hold contracts with NHS England and only access the platform to initiate database queries and statistical models; all database activity is logged; only aggregate statistical outputs leave the platform environment following best practice for anonymisation of results such as statistical disclosure control for low cell counts.^2^

The OpenSAFELY research platform adheres to the obligations of the UK General Data Protection Regulation (GDPR) and the Data Protection Act 2018. In March 2020, the Secretary of State for Health and Social Care used powers under the UK Health Service (Control of Patient Information) Regulations 2002 (COPI) to require organisations to process confidential patient information for the purposes of protecting public health, providing healthcare services to the public and monitoring and managing the COVID-19 outbreak and incidents of exposure; this sets aside the requirement for patient consent.^3^ This was extended in July 2022 for the NHS England OpenSAFELY COVID-19 research platform.^4^ In some cases of data sharing, the common law duty of confidence is met using, for example, patient consent or support from the Health Research Authority Confidentiality Advisory Group.^5^

Taken together, these provide the legal bases to link patient datasets on the OpenSAFELY platform. GP practices, from which the primary care data are obtained, are required to share relevant health information to support the public health response to the pandemic, and have been informed of the OpenSAFELY analytics platform.

### Results

#### Variant-specific analysis

The follow-up time, number of events and estimates of booster effectiveness during each variant era are summarised in eTable 1; cumulative incidence curves are given in eFigure 1.

Estimated adjusted hazard ratios (aHRs) for positive SARS-CoV-2 test during the period 1-14 days since booster dose were 0.53 (0.52, 0.54), 0.61 (0.60, 0.62) and 0.56 (0.55, 0.57) during the delta, transition and omicron eras respectively. By 15-42 since booster dose, the differences in aHRs had widened, with aHRs lowest during the delta era (0.23 (0.22, 0.24)) and highest during the omicron era (0.62 (0.61, 0.62)). This trend persisted for as long as there was sufficient follow-up to estimate era-specific booster effectiveness (up to 70 days for delta and 107 days for the transition era).

Estimated aHRs for COVID-19 hospitalisation were similar in the delta and transition eras (0.15 (0.13, 0.18) and 0.14 (0.11, 0.17) respectively during the period 15-42 days since booster dose), and higher in the omicron era (0.27 (0.23, 0.32) during the same time period). A similar trend was observed for non-COVID-19 death (estimated booster effectiveness during the period 15-42 days since booster dose was 0.21 (0.19, 0.23), 0.17 (0.14, 0.20) and 0.40 (0.30, 0.53) during the delta, transition and omicron eras, respectively). Estimates for COVID-19 death and CVD- and cancer-related non-COVID-deaths were imprecise and it is therefore difficult to comment on differences between the variant eras.

Estimated aHRs for fracture ranged between 0.68 (0.65, 0.72) and 0.84 (0.69, 1.03) during the delta era, 0.62 (0.47, 0.83) and 0.81 (0.73, 0.89) during the transition, and 0.82 (0.77, 0.88) and 0.89 (0.83, 0.95) during the omicron era.

#### Age subgroups

The follow-up time, number of events and estimates of booster effectiveness in the age subgroups are summarised in eTable 2; cumulative incidence curves and adjusted HRs are given in eFigure 2. The cumulative incidence of positive SARS-CoV-2 test was highest in the youngest subgroup (18-49 years), while the cumulative incidence of all other outcomes was highest in the oldest subgroup (80+ years).

Estimated aHRs for positive SARS-CoV-2 test during the period 1-14 days since booster dose were similar across the age subgroups (ranging between 0.56 (0.51, 0.62) and 0.59 (0.58, 0.60)). In subsequent time periods, estimated aHRs were lowest in the 80+ years subgroup (waning from 0.18 (0.16, 0.21) during days 15-42 after booster dose, to 0.87 (0.71, 1.06) 155-182 days after booster dose), and highest in the 18-49 years subgroup (waning from 0.56 (0.55, 0.56) during days 15-42 after booster dose, to 1.93 (1.75, 2.13) 155-182 days after booster dose).

Estimated aHRs were greater for COVID-19 hospitalisation and non-COVID-19 death compared to positive SARS-CoV-2 test, but showed similar patterns across the age subgroups (with the exception that estimates in the 65-79 and 80+ subgroups were more similar to each other for these outcomes compared to SARS-CoV-2 test). Estimates for COVID-19 death, CVD- and cancer-related non-COVID-19 death were imprecise and it is therefore difficult to comment on differences across the age subgroups. Estimated aHRs for fracture were similar across the age subgroups and across time since booster dose.

#### Prior SARS-CoV-2 infection subgroups

The follow-up time, number of events and estimates of booster effectiveness in the prior SARS-CoV-2 infection subgroups are summarised in eTable 3; cumulative incidence curves and aHRs are given in eFigure 3.

Estimated aHRs for positive SARS-CoV-2 test during the period 1-14 days since booster dose were similar across those with and without documented prior infection (0.60 (0.58, 0.62) and 0.61 (0.61, 0.62) respectively). In subsequent time periods, estimated aHRs were lower in those with documented prior infection (waning from 0.45 (0.43, 0.46) during days 15-42 after booster dose, to 1.34 (1.09, 1.64) 155-182 days after booster dose), compared to those without documented prior infection (waning from 0.58 (0.58, 0.59) during days 15-42 after booster dose, to 1.98 (1.84, 2.14) 155-182 days after booster dose).

Estimated aHRs for COVID-19 hospitalisation and non-COVID-19 death were also similar across the prior infection subgroups 1-14 days after booster dose, but then were typically slightly higher in those without documented prior infection across all subsequent time periods. Estimates for COVID-19 death were imprecise and it is therefore difficult to comment on differences across the prior infection subgroups. Estimated aHRs for fracture increased slightly across time periods, and were slightly lower in those without documented prior infection (0.68 (0.65, 0.72) and 0.80 (0.74, 0.86) during days 1-14 and 155-182 respectively) compared to those with (0.81 (0.71, 0.92) and 0.99 (0.84, 1.16) during days 1-14 and 155-182 respectively).

#### Clinical vulnerability subgroups

The follow-up time, number of events and estimates of booster effectiveness in the clinical vulnerability subgroups are summarised in eTable 4; cumulative incidence curves and adjusted HRs are given in eFigure 4. The cumulative incidence of SARS-CoV-2 test was highest in the “not clinically at risk” subgroup, while the cumulative incidence of all other outcomes was highest in the unboosted group of the “clinically extremely vulnerable” subgroup.

Estimated aHRs for positive SARS-CoV-2 test were similar across the clinical vulnerability subgroups during days 1-14 after booster dose (0.61 (0.60, 0.61), 0.64 (0.62, 0.65) and 0.63 (0.60, 0.65) in the “not clinically at-risk”, “clinically at-risk” and “clinically extremely vulnerable” subgroups respectively). Across all other comparison periods, estimated aHRs were lowest in the “clinically extremely vulnerable” subgroup, and highest in the “not clinically at-risk” subgroup. Within the subgroup, the aHRs waned over time, starting at 0.58 (0.57, 0.58), 0.51 (0.50, 0.53) and 0.44 (0.42, 0.46) during days 15-42 and waning to 2.10 (1.92, 2.31), 1.60 (1.37, 1.87) and 1.40 (1.18, 1.66) during days 155-182 in the “not clinically at-risk”, “clinically at-risk” and “clinically extremely vulnerable” subgroups respectively.

#### Primary course subgroups

The follow-up time, number of events and estimates of booster effectiveness in the clinical vulnerability subgroups are summarised in eTable 5; cumulative incidence curves and adjusted HRs are given in eFigure 5. The cumulative incidence of all events in the boosted group were similar regardless of primary course brand. However, in the unboosted group, the cumulative incidence of SARS-CoV-2 test, COVID-19 hospitalisation and COVID-19 death was slightly higher in the subgroup who received ChAdOx1 as their primary course, while the cumulative incidence of non-COVID-19 deaths (all, CVD-related and cancer-related) was slightly higher in the subgroup who received BNT162b2 as their primary course. Cumulative incidence of fracture was similar in both unboosted subgroups regardless of primary course brand. There was little evidence of differences in aHRs between the primary course subgroups.

#### No evidence of cancer in the 5 years prior to the trial start date

eTable 6 gives period-specific aHRs for boosted vs unboosted in those with no evidence of cancer in the 5 years prior to the trial start date (referred to from hereon as the “non-cancer subgroup”, which comprised 15,536,214 individuals; 95.1% and 94.4% of the unboosted and boosted individuals respectively in the full cohort). eFigure 6 compares the cumulative incidence and aHRs in the main cohort and non-cancer subgroup. The cumulative incidences were higher in the main cohort compared to the non-cancer subgroup for all outcomes, except for positive SARS-CoV-2 test and fracture for which there was little difference. Estimated aHRs were very similar in the main cohort and non-cancer subgroup.

eTable 1. Period-specific adjusted hazard ratios (Cox model), follow-up split by variant era.

| **Variant and days since booster** | **Positive SARS-CoV-2 test** | **COVID-19 hospitalisation** | **COVID-19 death** | **Non-COVID-19 death** | **CVD-related non-COVID-19 death** | **Cancer-related non-COVID-19 death** | **Fracture*** |
| --- | --- | --- | --- | --- | --- | --- | --- |
| **Delta variant** |  |  |  |  |  |  |  |
| 1-14 | 0.53 (0.52, 0.54) | 0.29 (0.26, 0.33) | 0.26 (0.16, 0.41) | 0.31 (0.28, 0.35) | 0.41 (0.34, 0.49) | 0.16 (0.12, 0.22) | 0.68 (0.65, 0.72) |
| 15-42 | 0.23 (0.22, 0.24) | 0.15 (0.13, 0.18) | 0.14 (0.10, 0.19) | 0.21 (0.19, 0.23) | 0.24 (0.20, 0.28) | 0.15 (0.12, 0.19) | 0.73 (0.68, 0.79) |
| 43-70 | 0.36 (0.32, 0.41) | 0.17 (0.12, 0.26) | 0.08 (0.03, 0.19) | 0.15 (0.11, 0.18) | 0.11 (0.07, 0.19) | 0.18 (0.12, 0.27) | 0.84 (0.69, 1.03) |
| **Delta-omicron trans.** |  |  |  |  |  |  |  |
| 1-14 | 0.61 (0.60, 0.62) | 0.30 (0.24, 0.36) |  | 0.20 (0.15, 0.28) | 0.22 (0.11, 0.40) | 0.16 (0.06, 0.47) | 0.75 (0.69, 0.81) |
| 15-42 | 0.42 (0.41, 0.43) | 0.14 (0.11, 0.17) | 0.08 (0.04, 0.16) | 0.17 (0.14, 0.20) | 0.25 (0.18, 0.34) | 0.12 (0.08, 0.18) | 0.81 (0.73, 0.89) |
| 43-70 | 0.63 (0.61, 0.65) | 0.16 (0.13, 0.20) | 0.04 (0.02, 0.08) | 0.15 (0.13, 0.17) | 0.17 (0.13, 0.23) | 0.11 (0.08, 0.15) | 0.64 (0.56, 0.74) |
| 71-98 | 0.71 (0.67, 0.75) | 0.21 (0.15, 0.29) |  | 0.12 (0.09, 0.16) | 0.15 (0.09, 0.25) | 0.11 (0.06, 0.19) | 0.62 (0.47, 0.83) |
| 99-126 | 0.97 (0.62, 1.52) |  |  |  |  |  |  |
| **Omicron variant** |  |  |  |  |  |  |  |
| 1-14 | 0.56 (0.55, 0.57) | 0.42 (0.28, 0.62) |  | 0.44 (0.19, 1.01) |  |  | 0.86 (0.73, 1.01) |
| 15-42 | 0.62 (0.61, 0.62) | 0.27 (0.23, 0.32) | 0.26 (0.10, 0.66) | 0.40 (0.30, 0.53) | 0.39 (0.21, 0.73) | 0.19 (0.09, 0.41) | 0.89 (0.82, 0.95) |
| 43-70 | 0.97 (0.95, 0.98) | 0.31 (0.27, 0.35) | 0.10 (0.04, 0.21) | 0.23 (0.19, 0.28) | 0.18 (0.11, 0.27) | 0.15 (0.10, 0.23) | 0.85 (0.79, 0.91) |
| 71-98 | 1.30 (1.28, 1.32) | 0.34 (0.30, 0.39) | 0.10 (0.06, 0.16) | 0.19 (0.17, 0.22) | 0.21 (0.16, 0.28) | 0.17 (0.13, 0.22) | 0.88 (0.82, 0.94) |
| 99-126 | 1.59 (1.56, 1.63) | 0.44 (0.39, 0.50) | 0.14 (0.08, 0.23) | 0.24 (0.21, 0.28) | 0.28 (0.21, 0.36) | 0.28 (0.22, 0.36) | 0.85 (0.79, 0.90) |
| 127-154 | 1.71 (1.64, 1.79) | 0.49 (0.43, 0.56) | 0.07 (0.04, 0.15) | 0.28 (0.25, 0.33) | 0.36 (0.27, 0.49) | 0.38 (0.30, 0.49) | 0.82 (0.77, 0.88) |
| 155-182 | 1.68 (1.56, 1.80) | 0.58 (0.51, 0.67) | 0.26 (0.16, 0.41) | 0.37 (0.32, 0.43) | 0.42 (0.31, 0.56) | 0.36 (0.28, 0.48) | 0.89 (0.83, 0.95) |

*Estimates for fracture are unadjusted as the adjusted models failed due to memory constraints.


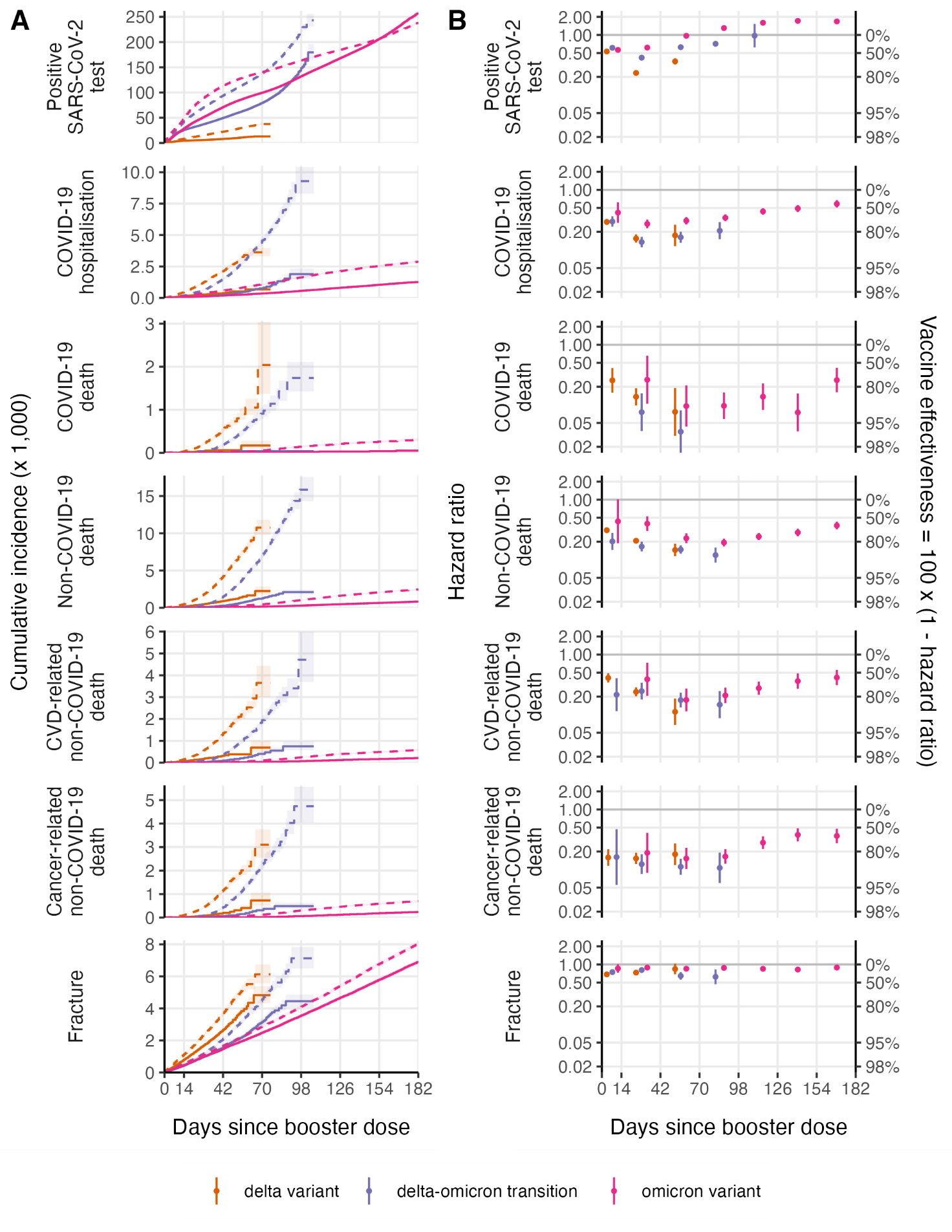


eFigure 1. Follow-up split by variant era: (A) Kaplan-Meier estimates of cumulative incidence in matched boosted and unboosted treatment groups, without further adjustment for confounders. Dashed line = unboosted, solid line = boosted. (B) Cox hazard ratio estimates with adjustment for confounders.

eTable 2. Period-specific adjusted hazard ratios (Cox model), across age subgroups.

| **Age group and days since booster** | **Positive SARS-CoV-2 test** | **COVID-19 hospitalisation** | **COVID-19 death** | **Non-COVID-19 death** | **CVD-related non-COVID-19 death** | **Cancer-related non-COVID-19 death** | **Fracture** |
| --- | --- | --- | --- | --- | --- | --- | --- |
| **18-49 years** |  |  |  |  |  |  |  |
| 1-14 | 0.59 (0.58, 0.59) | 0.32 (0.26, 0.39) |  | 0.29 (0.18, 0.46) | 0.69 (0.30, 1.56) |  | 0.74 (0.68, 0.81) |
| 15-42 | 0.56 (0.55, 0.56) | 0.27 (0.23, 0.32) | 0.24 (0.08, 0.73) | 0.46 (0.34, 0.64) | 0.38 (0.17, 0.86) | 0.25 (0.10, 0.61) | 0.88 (0.82, 0.94) |
| 43-70 | 0.91 (0.90, 0.93) | 0.46 (0.39, 0.55) |  | 0.43 (0.31, 0.61) | 0.40 (0.17, 0.91) | 0.16 (0.06, 0.48) | 0.83 (0.77, 0.90) |
| 71-98 | 1.25 (1.23, 1.27) | 0.55 (0.45, 0.66) |  | 0.48 (0.33, 0.70) | 0.23 (0.09, 0.62) | 0.62 (0.29, 1.35) | 0.87 (0.80, 0.94) |
| 99-126 | 1.55 (1.51, 1.60) | 0.62 (0.51, 0.75) | 1.04 (0.21, 5.11) | 0.54 (0.36, 0.81) | 0.50 (0.21, 1.18) | 0.60 (0.24, 1.48) | 0.81 (0.75, 0.87) |
| 127-154 | 1.70 (1.61, 1.81) | 0.73 (0.57, 0.93) | 0.00 (0.00, 0.00) | 0.55 (0.35, 0.85) | 0.82 (0.29, 2.27) | 0.80 (0.31, 2.07) | 0.81 (0.75, 0.87) |
| 155-182 | 1.93 (1.75, 2.13) | 0.86 (0.66, 1.12) |  | 0.61 (0.40, 0.94) | 0.54 (0.21, 1.38) | 0.45 (0.15, 1.32) | 0.86 (0.79, 0.93) |
| **50-64 years** |  |  |  |  |  |  |  |
| 1-14 | 0.59 (0.58, 0.60) | 0.34 (0.27, 0.41) | 0.23 (0.08, 0.69) | 0.28 (0.22, 0.35) | 0.36 (0.24, 0.56) | 0.11 (0.05, 0.24) | 0.78 (0.71, 0.85) |
| 15-42 | 0.48 (0.47, 0.49) | 0.19 (0.15, 0.23) | 0.31 (0.16, 0.60) | 0.28 (0.23, 0.34) | 0.38 (0.26, 0.57) | 0.24 (0.16, 0.36) | 0.82 (0.74, 0.90) |
| 43-70 | 0.81 (0.78, 0.83) | 0.24 (0.19, 0.30) | 0.08 (0.03, 0.28) | 0.23 (0.18, 0.30) | 0.19 (0.10, 0.35) | 0.20 (0.12, 0.31) | 0.91 (0.80, 1.04) |
| 71-98 | 1.21 (1.16, 1.25) | 0.37 (0.28, 0.48) |  | 0.26 (0.20, 0.34) | 0.40 (0.23, 0.70) | 0.21 (0.13, 0.33) | 0.88 (0.76, 1.02) |
| 99-126 | 1.68 (1.61, 1.75) | 0.46 (0.35, 0.60) |  | 0.35 (0.27, 0.47) | 0.41 (0.23, 0.71) | 0.26 (0.16, 0.43) | 0.93 (0.80, 1.08) |
| 127-154 | 1.89 (1.74, 2.06) | 0.62 (0.48, 0.81) |  | 0.36 (0.27, 0.49) | 0.51 (0.27, 0.94) | 0.70 (0.43, 1.13) | 0.84 (0.72, 0.98) |
| 155-182 | 1.59 (1.38, 1.83) | 0.56 (0.40, 0.79) | 0.67 (0.21, 2.12) | 0.53 (0.40, 0.72) | 0.82 (0.44, 1.51) | 0.43 (0.27, 0.70) | 0.91 (0.78, 1.07) |
| **65-79 years** |  |  |  |  |  |  |  |
| 1-14 | 0.56 (0.54, 0.58) | 0.29 (0.25, 0.34) | 0.21 (0.10, 0.44) | 0.35 (0.29, 0.41) | 0.47 (0.36, 0.62) | 0.22 (0.14, 0.32) | 0.72 (0.66, 0.78) |
| 15-42 | 0.23 (0.22, 0.25) | 0.13 (0.10, 0.16) | 0.12 (0.08, 0.18) | 0.18 (0.16, 0.21) | 0.26 (0.20, 0.33) | 0.15 (0.11, 0.20) | 0.77 (0.70, 0.86) |
| 43-70 | 0.69 (0.64, 0.73) | 0.16 (0.13, 0.21) | 0.07 (0.04, 0.15) | 0.12 (0.10, 0.15) | 0.12 (0.08, 0.18) | 0.10 (0.07, 0.15) | 0.58 (0.48, 0.70) |
| 71-98 | 0.90 (0.83, 0.98) | 0.21 (0.16, 0.27) | 0.05 (0.02, 0.13) | 0.13 (0.10, 0.17) | 0.15 (0.09, 0.25) | 0.11 (0.06, 0.17) | 0.86 (0.68, 1.09) |
| 99-126 | 1.27 (1.15, 1.41) | 0.34 (0.26, 0.46) | 0.13 (0.05, 0.33) | 0.22 (0.17, 0.28) | 0.23 (0.14, 0.39) | 0.27 (0.19, 0.40) | 0.83 (0.64, 1.08) |
| 127-154 | 1.61 (1.45, 1.79) | 0.37 (0.28, 0.49) |  | 0.25 (0.20, 0.33) | 0.26 (0.14, 0.46) | 0.30 (0.20, 0.45) | 0.76 (0.58, 1.00) |
| 155-182 | 1.72 (1.38, 2.16) | 0.37 (0.27, 0.49) | 0.26 (0.11, 0.63) | 0.26 (0.19, 0.36) | 0.32 (0.18, 0.59) | 0.25 (0.15, 0.42) | 0.79 (0.60, 1.03) |
| **80+ years** |  |  |  |  |  |  |  |
| 1-14 | 0.56 (0.51, 0.62) | 0.29 (0.24, 0.36) | 0.32 (0.15, 0.67) | 0.29 (0.24, 0.34) | 0.35 (0.27, 0.46) | 0.12 (0.07, 0.22) | 0.59 (0.53, 0.65) |
| 15-42 | 0.18 (0.16, 0.21) | 0.15 (0.12, 0.19) | 0.10 (0.06, 0.17) | 0.21 (0.19, 0.24) | 0.24 (0.19, 0.29) | 0.12 (0.09, 0.17) | 0.65 (0.58, 0.72) |
| 43-70 | 0.25 (0.21, 0.29) | 0.15 (0.12, 0.20) | 0.04 (0.02, 0.10) | 0.18 (0.15, 0.21) | 0.19 (0.14, 0.25) | 0.17 (0.12, 0.23) | 0.67 (0.56, 0.80) |
| 71-98 | 0.47 (0.41, 0.53) | 0.22 (0.17, 0.29) | 0.10 (0.05, 0.19) | 0.15 (0.12, 0.19) | 0.18 (0.12, 0.27) | 0.13 (0.08, 0.20) | 0.57 (0.45, 0.72) |
| 99-126 | 0.52 (0.44, 0.61) | 0.32 (0.24, 0.42) | 0.09 (0.04, 0.20) | 0.19 (0.15, 0.24) | 0.25 (0.17, 0.36) | 0.29 (0.18, 0.46) | 0.71 (0.54, 0.94) |
| 127-154 | 0.59 (0.49, 0.71) | 0.30 (0.22, 0.41) | 0.07 (0.03, 0.21) | 0.24 (0.18, 0.31) | 0.35 (0.22, 0.55) | 0.26 (0.15, 0.43) | 0.63 (0.46, 0.85) |
| 155-182 | 0.87 (0.71, 1.06) | 0.58 (0.45, 0.76) | 0.19 (0.10, 0.38) | 0.33 (0.26, 0.42) | 0.34 (0.22, 0.52) | 0.45 (0.27, 0.75) | 0.82 (0.60, 1.13) |


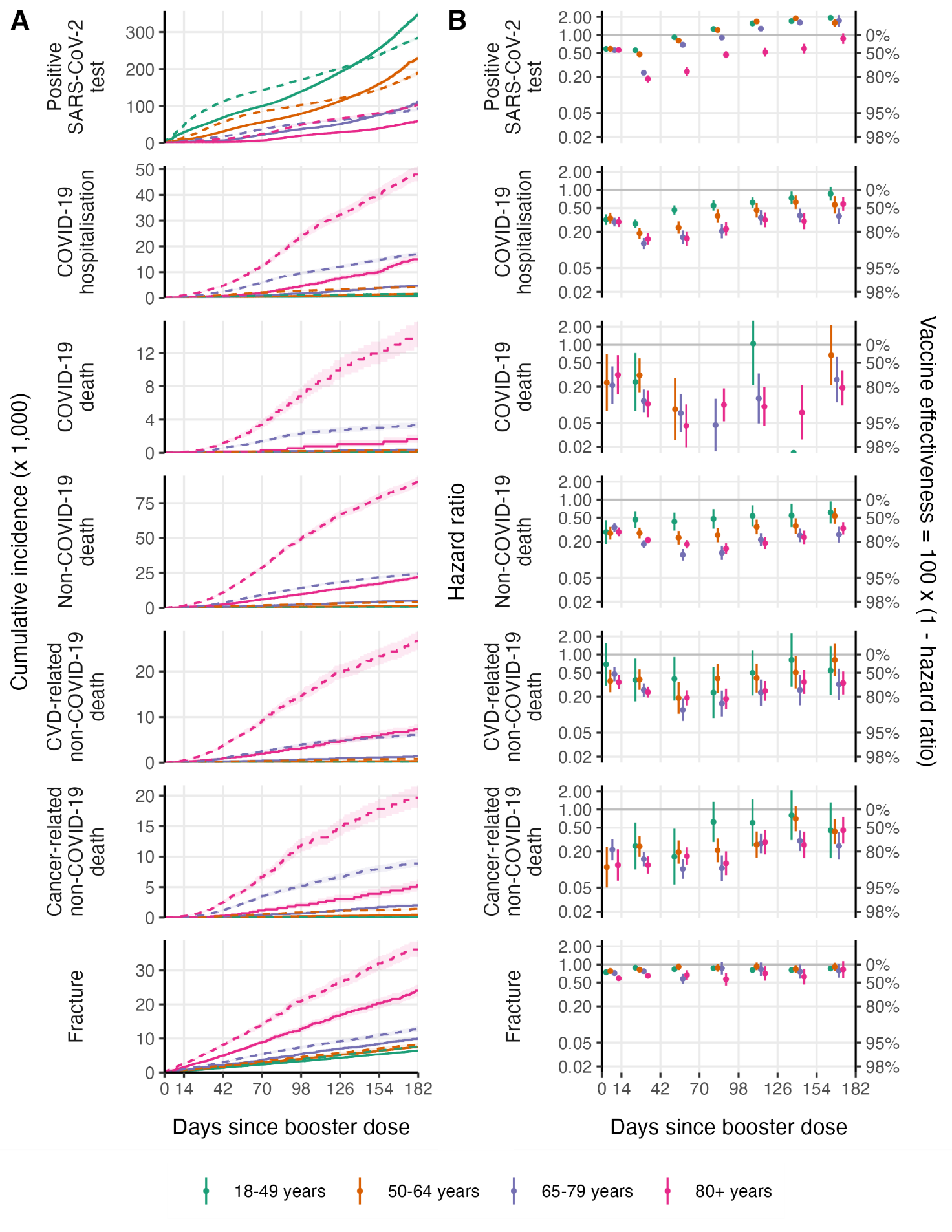


eFigure 2. Age subgroups: (A) Kaplan-Meier estimates of cumulative incidence in matched boosted and unboosted treatment groups, without further adjustment for confounders. Dashed line = unboosted, solid line = boosted. (B) Cox hazard ratio estimates for boosted vs unboosted, with adjustment for confounders.

eTable 3. Period-specific adjusted hazard ratios (Cox model), across prior infection subgroups.

| **Prior infection subgroup and days since booster** | **Positive SARS-CoV-2 test** | **COVID-19 hospitalisation** | **COVID-19 death** | **Non-COVID-19 death** | **CVD-related non-COVID-19 death** | **Cancer-related non-COVID-19 death** | **Fracture** |
| --- | --- | --- | --- | --- | --- | --- | --- |
| **No prior SARS-CoV-2 infection** | |  |  |  |  |  |  |
| 1-14 | 0.61 (0.61, 0.62) | 0.30 (0.27, 0.33) | 0.23 (0.14, 0.37) | 0.29 (0.26, 0.33) | 0.38 (0.32, 0.45) | 0.15 (0.11, 0.21) | 0.68 (0.65, 0.72) |
| 15-42 | 0.58 (0.58, 0.59) | 0.17 (0.15, 0.19) | 0.13 (0.09, 0.17) | 0.20 (0.18, 0.22) | 0.24 (0.20, 0.27) | 0.14 (0.12, 0.17) | 0.76 (0.72, 0.80) |
| 43-70 | 0.97 (0.96, 0.98) | 0.23 (0.21, 0.26) | 0.05 (0.03, 0.08) | 0.16 (0.14, 0.18) | 0.16 (0.13, 0.20) | 0.13 (0.10, 0.16) | 0.76 (0.72, 0.81) |
| 71-98 | 1.37 (1.35, 1.39) | 0.31 (0.27, 0.35) | 0.08 (0.05, 0.13) | 0.16 (0.14, 0.19) | 0.17 (0.13, 0.23) | 0.14 (0.11, 0.18) | 0.78 (0.73, 0.84) |
| 99-126 | 1.82 (1.77, 1.86) | 0.43 (0.38, 0.49) | 0.14 (0.08, 0.23) | 0.23 (0.20, 0.26) | 0.26 (0.20, 0.34) | 0.27 (0.21, 0.34) | 0.78 (0.73, 0.84) |
| 127-154 | 2.01 (1.92, 2.10) | 0.48 (0.41, 0.55) | 0.06 (0.03, 0.14) | 0.27 (0.23, 0.32) | 0.36 (0.27, 0.48) | 0.37 (0.28, 0.47) | 0.76 (0.71, 0.82) |
| 155-182 | 1.98 (1.84, 2.14) | 0.58 (0.50, 0.67) | 0.25 (0.15, 0.40) | 0.35 (0.30, 0.41) | 0.41 (0.30, 0.55) | 0.34 (0.25, 0.45) | 0.80 (0.74, 0.86) |
| **Prior SARS-CoV-2 infection** | |  |  |  |  |  |  |
| 1-14 | 0.60 (0.58, 0.62) | 0.34 (0.23, 0.50) |  | 0.27 (0.18, 0.41) | 0.39 (0.21, 0.69) | 0.19 (0.07, 0.50) | 0.81 (0.71, 0.92) |
| 15-42 | 0.45 (0.43, 0.46) | 0.28 (0.20, 0.39) |  | 0.25 (0.18, 0.35) | 0.27 (0.15, 0.49) | 0.19 (0.09, 0.40) | 0.89 (0.79, 1.00) |
| 43-70 | 0.73 (0.71, 0.76) | 0.35 (0.24, 0.50) | 0.27 (0.08, 0.98) | 0.19 (0.13, 0.28) | 0.10 (0.04, 0.24) | 0.19 (0.09, 0.39) | 0.82 (0.71, 0.94) |
| 71-98 | 1.01 (0.97, 1.06) | 0.47 (0.31, 0.70) |  | 0.21 (0.13, 0.32) | 0.29 (0.12, 0.69) | 0.20 (0.09, 0.44) | 0.97 (0.84, 1.13) |
| 99-126 | 1.32 (1.24, 1.41) | 0.50 (0.33, 0.78) | 0.00 (0.00, 0.00) | 0.27 (0.15, 0.49) | 0.19 (0.05, 0.67) |  | 0.86 (0.74, 1.01) |
| 127-154 | 1.51 (1.33, 1.70) | 0.67 (0.43, 1.06) |  | 0.24 (0.14, 0.42) |  | 0.21 (0.07, 0.63) | 0.83 (0.71, 0.96) |
| 155-182 | 1.34 (1.09, 1.64) | 0.65 (0.37, 1.15) |  | 0.38 (0.22, 0.66) | 0.22 (0.07, 0.70) | 0.36 (0.12, 1.04) | 0.99 (0.84, 1.16) |


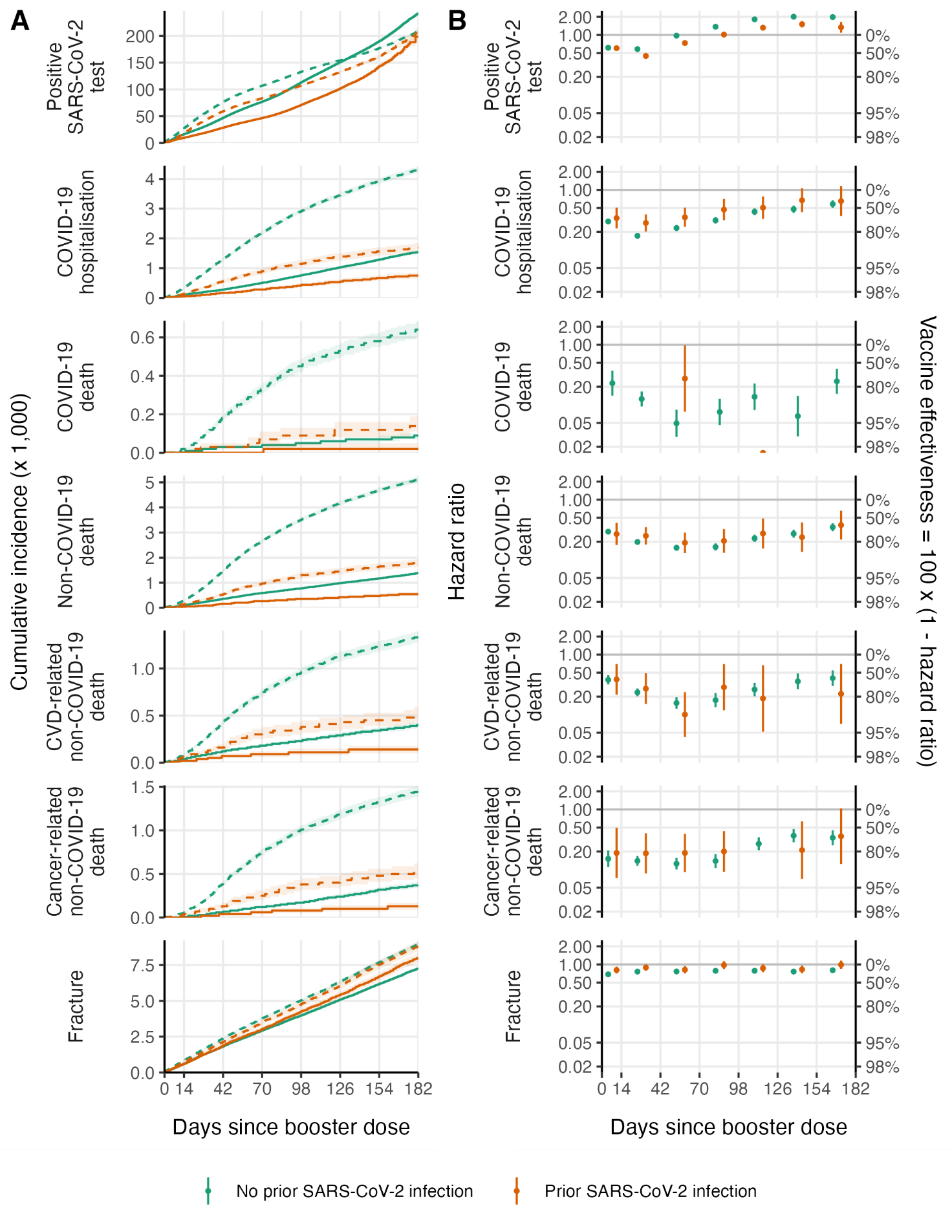


eFigure 3. Prior infection subgroups: (A) Kaplan-Meier estimates of cumulative incidence in matched boosted and unboosted treatment groups, without further adjustment for confounders. Dashed line = unboosted, solid line = boosted. (B) Cox hazard ratio estimates for confounders.

eTable 4. Period-specific adjusted hazard ratios (Cox model), across risk subgroups.

| **Risk subgroup and days since booster** | **Positive SARS-CoV-2 test** | **COVID-19 hospitalisation** | **COVID-19 death** | **Non-COVID-19 death** | **CVD-related non-COVID-19 death** | **Cancer-related non-COVID-19 death** | **Fracture** |
| --- | --- | --- | --- | --- | --- | --- | --- |
| **Not clinically at-risk** | |  |  |  |  |  |  |
| 1-14 | 0.61 (0.60, 0.61) | 0.28 (0.23, 0.34) | 0.49 (0.14, 1.68) | 0.36 (0.28, 0.45) | 0.59 (0.41, 0.86) | 0.11 (0.04, 0.28) | 0.73 (0.69, 0.78) |
| 15-42 | 0.58 (0.57, 0.58) | 0.20 (0.16, 0.23) |  | 0.19 (0.15, 0.23) | 0.28 (0.19, 0.41) | 0.05 (0.03, 0.10) | 0.85 (0.80, 0.90) |
| 43-70 | 0.97 (0.96, 0.99) | 0.32 (0.26, 0.38) | 0.07 (0.02, 0.22) | 0.17 (0.13, 0.22) | 0.23 (0.13, 0.39) | 0.09 (0.05, 0.16) | 0.81 (0.75, 0.87) |
| 71-98 | 1.35 (1.33, 1.38) | 0.36 (0.29, 0.45) |  | 0.20 (0.15, 0.26) | 0.23 (0.13, 0.42) | 0.14 (0.08, 0.24) | 0.85 (0.79, 0.92) |
| 99-126 | 1.76 (1.72, 1.81) | 0.52 (0.42, 0.64) | 0.24 (0.09, 0.69) | 0.26 (0.19, 0.35) | 0.26 (0.14, 0.49) | 0.17 (0.10, 0.29) | 0.80 (0.74, 0.86) |
| 127-154 | 2.01 (1.91, 2.13) | 0.59 (0.45, 0.76) |  | 0.23 (0.16, 0.33) | 0.67 (0.36, 1.24) | 0.22 (0.12, 0.41) | 0.80 (0.74, 0.86) |
| 155-182 | 2.10 (1.92, 2.31) | 0.58 (0.44, 0.75) | 0.18 (0.05, 0.66) | 0.31 (0.23, 0.43) | 0.50 (0.28, 0.88) | 0.15 (0.08, 0.28) | 0.85 (0.78, 0.92) |
| **Clinically at-risk** |  |  |  |  |  |  |  |
| 1-14 | 0.64 (0.62, 0.65) | 0.30 (0.25, 0.35) | 0.09 (0.03, 0.26) | 0.27 (0.23, 0.32) | 0.38 (0.29, 0.49) | 0.14 (0.08, 0.23) | 0.67 (0.62, 0.72) |
| 15-42 | 0.51 (0.50, 0.53) | 0.14 (0.11, 0.16) | 0.08 (0.05, 0.14) | 0.19 (0.17, 0.22) | 0.23 (0.19, 0.29) | 0.11 (0.08, 0.16) | 0.68 (0.62, 0.74) |
| 43-70 | 0.88 (0.85, 0.91) | 0.21 (0.17, 0.25) | 0.04 (0.01, 0.10) | 0.13 (0.11, 0.16) | 0.11 (0.07, 0.16) | 0.10 (0.07, 0.14) | 0.73 (0.64, 0.83) |
| 71-98 | 1.26 (1.21, 1.32) | 0.25 (0.20, 0.31) | 0.07 (0.03, 0.17) | 0.14 (0.12, 0.18) | 0.17 (0.11, 0.26) | 0.12 (0.08, 0.18) | 0.80 (0.69, 0.93) |
| 99-126 | 1.78 (1.69, 1.87) | 0.40 (0.32, 0.50) | 0.07 (0.02, 0.24) | 0.21 (0.16, 0.26) | 0.19 (0.13, 0.30) | 0.28 (0.18, 0.43) | 0.77 (0.66, 0.90) |
| 127-154 | 1.94 (1.77, 2.13) | 0.34 (0.27, 0.43) |  | 0.23 (0.18, 0.29) | 0.23 (0.14, 0.37) | 0.23 (0.15, 0.36) | 0.75 (0.63, 0.88) |
| 155-182 | 1.60 (1.37, 1.87) | 0.55 (0.43, 0.71) | 0.29 (0.13, 0.67) | 0.30 (0.23, 0.38) | 0.34 (0.21, 0.56) | 0.25 (0.14, 0.44) | 0.77 (0.66, 0.91) |
| **Clinically extremely vulnerable** | |  |  |  |  |  |  |
| 1-14 | 0.63 (0.60, 0.65) | 0.32 (0.27, 0.37) | 0.32 (0.18, 0.57) | 0.28 (0.24, 0.33) | 0.30 (0.22, 0.41) | 0.19 (0.12, 0.28) | 0.66 (0.59, 0.74) |
| 15-42 | 0.44 (0.42, 0.46) | 0.21 (0.18, 0.24) | 0.19 (0.13, 0.26) | 0.22 (0.19, 0.24) | 0.23 (0.19, 0.29) | 0.22 (0.17, 0.27) | 0.72 (0.65, 0.81) |
| 43-70 | 0.75 (0.72, 0.79) | 0.21 (0.18, 0.26) | 0.07 (0.04, 0.12) | 0.19 (0.17, 0.23) | 0.18 (0.14, 0.25) | 0.18 (0.14, 0.24) | 0.69 (0.58, 0.81) |
| 71-98 | 0.97 (0.91, 1.02) | 0.35 (0.29, 0.43) | 0.08 (0.04, 0.16) | 0.18 (0.15, 0.22) | 0.18 (0.12, 0.26) | 0.17 (0.12, 0.25) | 0.64 (0.52, 0.79) |
| 99-126 | 1.41 (1.31, 1.52) | 0.40 (0.32, 0.50) | 0.13 (0.07, 0.26) | 0.24 (0.20, 0.30) | 0.33 (0.23, 0.47) | 0.32 (0.23, 0.46) | 0.92 (0.73, 1.15) |
| 127-154 | 1.54 (1.40, 1.70) | 0.56 (0.46, 0.70) | 0.08 (0.03, 0.21) | 0.34 (0.28, 0.43) | 0.36 (0.22, 0.57) | 0.60 (0.42, 0.86) | 0.66 (0.52, 0.84) |
| 155-182 | 1.40 (1.18, 1.66) | 0.59 (0.47, 0.74) | 0.24 (0.13, 0.46) | 0.43 (0.34, 0.53) | 0.38 (0.24, 0.60) | 0.62 (0.42, 0.92) | 0.91 (0.71, 1.16) |


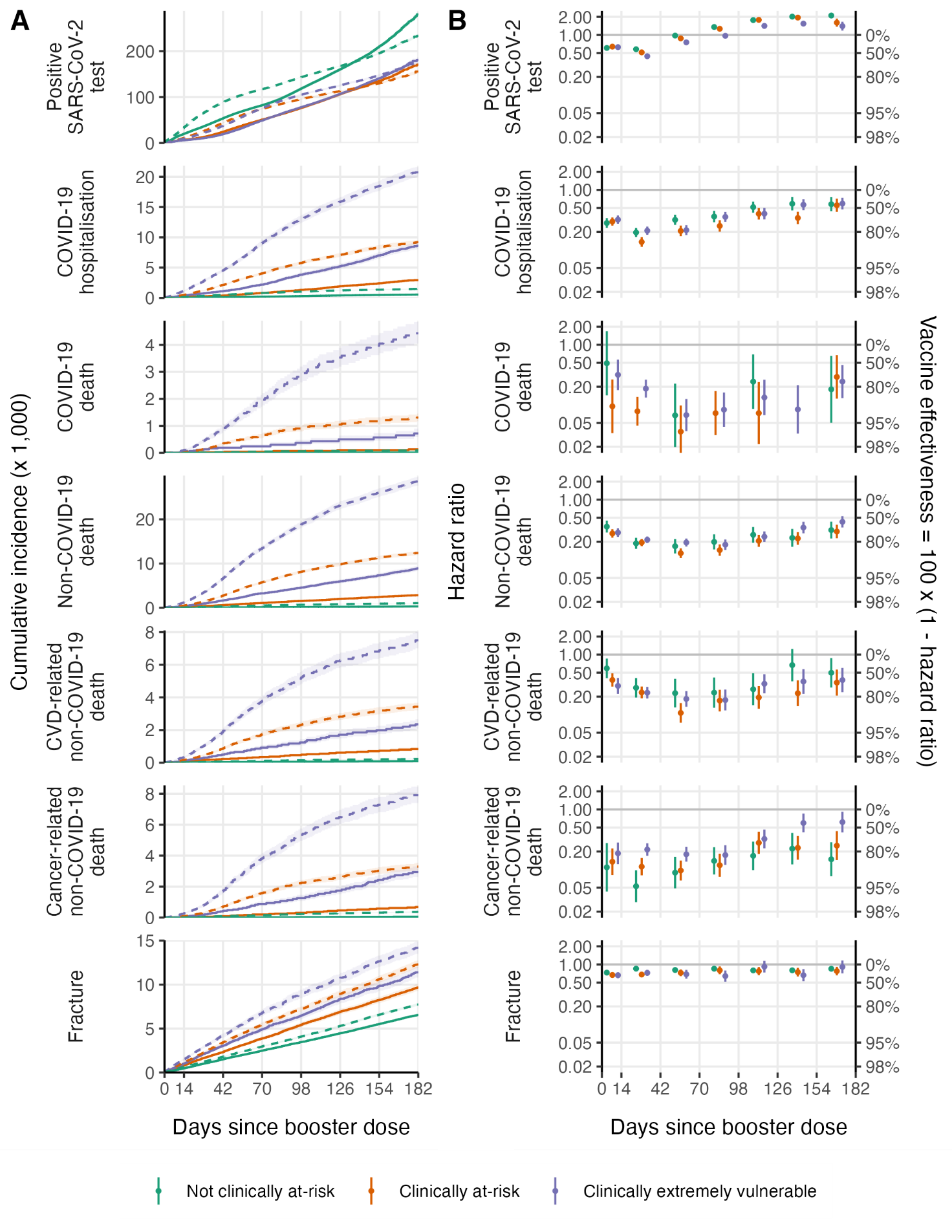


eFigure 4. Clinical risk subgroups: (A) Kaplan-Meier estimates of cumulative incidence in matched boosted and unboosted treatment groups, without further adjustment for confounders. Dashed line = unboosted, solid line = boosted. (B) Cox hazard ratio estimates for confounders.

eTable 5. Period-specific adjusted hazard ratios (Cox model), across primary course subgroups.

| Days since booster | Positive SARS-CoV-2 test | COVID-19 hospitalisation | COVID-19 death | Non-COVID-19 death | CVD-related non-COVID-19 death | Cancer-related non-COVID-19 death | Fracture |
| --- | --- | --- | --- | --- | --- | --- | --- |
| **BNT162b2** |  |  |  |  |  |  |  |
| 1-14 | 0.63 (0.60, 0.65) | 0.33 (0.26, 0.42) |  | 0.29 (0.23, 0.36) | 0.35 (0.23, 0.53) | 0.20 (0.11, 0.36) | 0.77 (0.69, 0.86) |
| 15-42 | 0.48 (0.46, 0.50) | 0.21 (0.16, 0.27) | 0.24 (0.13, 0.43) | 0.20 (0.17, 0.25) | 0.26 (0.18, 0.38) | 0.14 (0.09, 0.20) | 0.79 (0.69, 0.90) |
| 43-70 | 0.84 (0.81, 0.88) | 0.21 (0.16, 0.29) |  | 0.14 (0.11, 0.19) | 0.13 (0.07, 0.24) | 0.12 (0.08, 0.20) | 0.80 (0.66, 0.97) |
| 71-98 | 1.16 (1.10, 1.23) | 0.36 (0.27, 0.48) |  | 0.18 (0.13, 0.25) | 0.15 (0.08, 0.29) | 0.17 (0.10, 0.30) | 0.82 (0.65, 1.03) |
| 99-126 | 1.66 (1.54, 1.79) | 0.46 (0.33, 0.64) |  | 0.15 (0.10, 0.23) | 0.22 (0.10, 0.48) | 0.13 (0.06, 0.26) | 0.79 (0.62, 1.01) |
| 127-154 | 2.08 (1.88, 2.31) | 0.49 (0.36, 0.66) | 0.00 (0.00, 0.00) | 0.25 (0.17, 0.35) | 0.20 (0.09, 0.45) | 0.34 (0.20, 0.58) | 0.74 (0.57, 0.96) |
| 155-182 | 1.80 (1.52, 2.14) | 0.39 (0.26, 0.57) | 0.23 (0.07, 0.82) | 0.22 (0.14, 0.33) | 0.41 (0.16, 1.02) | 0.28 (0.14, 0.54) | 0.76 (0.58, 0.99) |
| **ChAdOx1-S** |  |  |  |  |  |  |  |
| 1-14 | 0.62 (0.61, 0.64) | 0.30 (0.26, 0.35) | 0.22 (0.10, 0.44) | 0.33 (0.28, 0.39) | 0.55 (0.42, 0.74) | 0.17 (0.11, 0.27) | 0.72 (0.66, 0.78) |
| 15-42 | 0.48 (0.47, 0.49) | 0.16 (0.13, 0.18) | 0.13 (0.09, 0.20) | 0.21 (0.18, 0.24) | 0.26 (0.20, 0.35) | 0.20 (0.15, 0.26) | 0.74 (0.68, 0.82) |
| 43-70 | 0.86 (0.83, 0.89) | 0.25 (0.21, 0.30) | 0.06 (0.03, 0.13) | 0.15 (0.12, 0.18) | 0.11 (0.07, 0.17) | 0.11 (0.08, 0.16) | 0.75 (0.65, 0.86) |
| 71-98 | 1.24 (1.19, 1.29) | 0.33 (0.27, 0.41) | 0.08 (0.03, 0.18) | 0.16 (0.13, 0.21) | 0.20 (0.13, 0.32) | 0.13 (0.08, 0.20) | 0.86 (0.74, 1.01) |
| 99-126 | 1.81 (1.72, 1.90) | 0.41 (0.34, 0.51) | 0.18 (0.07, 0.47) | 0.31 (0.25, 0.38) | 0.29 (0.19, 0.44) | 0.39 (0.28, 0.55) | 0.89 (0.76, 1.04) |
| 127-154 | 2.05 (1.88, 2.23) | 0.50 (0.41, 0.61) |  | 0.34 (0.27, 0.43) | 0.33 (0.19, 0.57) | 0.49 (0.34, 0.72) | 0.79 (0.67, 0.93) |
| 155-182 | 2.14 (1.77, 2.60) | 0.61 (0.48, 0.78) | 0.39 (0.17, 0.92) | 0.45 (0.35, 0.58) | 0.39 (0.23, 0.67) | 0.40 (0.25, 0.62) | 0.83 (0.70, 0.98) |


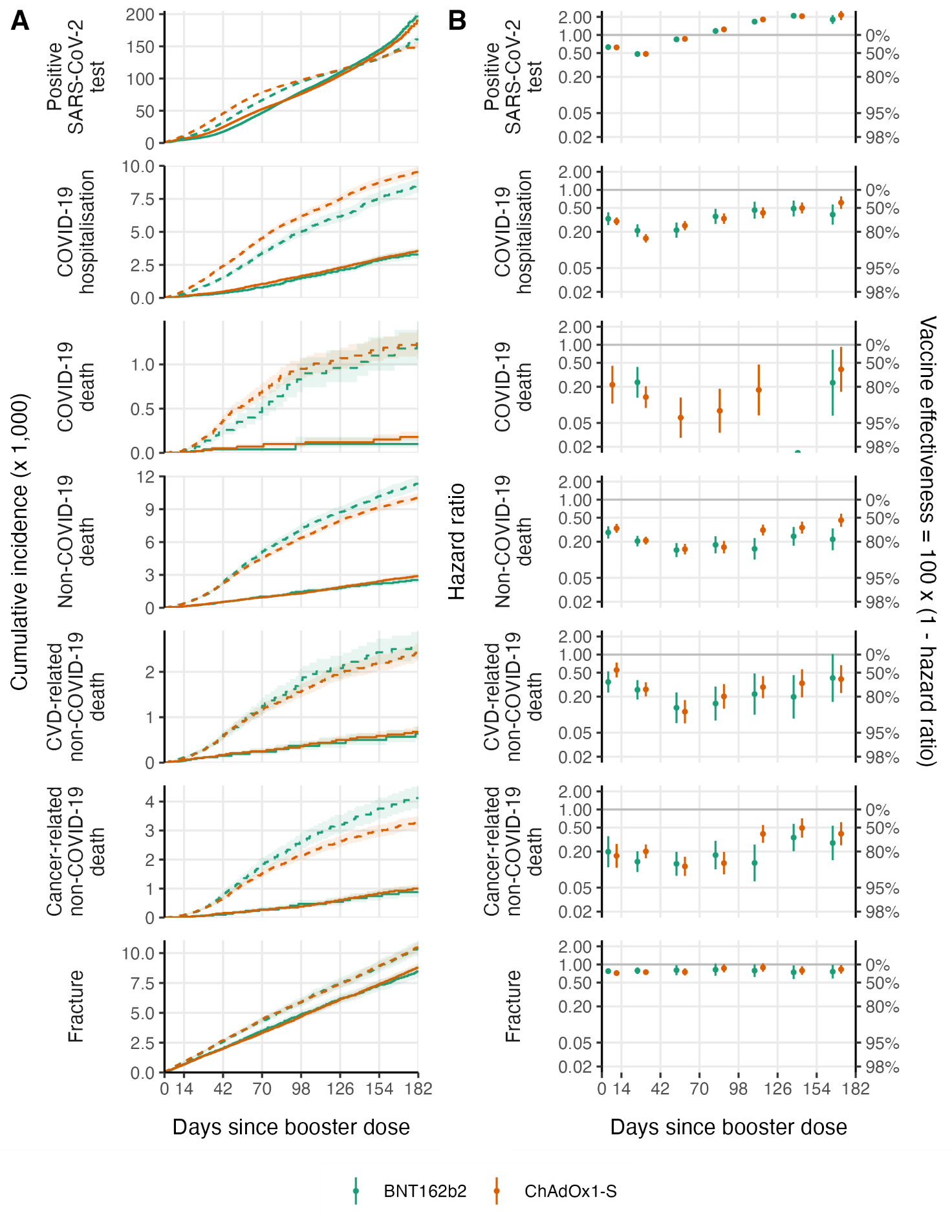


eFigure 5. Primary course subgroups: (A) Kaplan-Meier estimates of cumulative incidence in matched boosted and unboosted treatment groups, without further adjustment for confounders. Dashed line = unboosted, solid line = boosted. (B) Cox hazard ratio estimates for boosted vs unboosted, with adjustment for confounders.

eTable 6. Period-specific adjusted hazard ratios (Cox model) in those with no evidence of cancer in the 5 years prior to the trial start date.

| **Days since booster** | **Positive SARS-CoV-2 test** | **COVID-19 hospitalisation** | **COVID-19 death** | **Non-COVID-19 death** | **CVD-related non-COVID-19 death** | **Cancer-related non-COVID-19 death** | **Fracture** |
| --- | --- | --- | --- | --- | --- | --- | --- |
| 1-14 | 0.61 (0.61, 0.62) | 0.30 (0.27, 0.33) | 0.23 (0.14, 0.39) | 0.33 (0.30, 0.37) | 0.40 (0.33, 0.49) | 0.12 (0.06, 0.27) | 0.70 (0.67, 0.73) |
| 15-42 | 0.57 (0.56, 0.57) | 0.17 (0.15, 0.19) | 0.12 (0.09, 0.17) | 0.22 (0.20, 0.25) | 0.25 (0.21, 0.30) | 0.10 (0.07, 0.15) | 0.79 (0.75, 0.83) |
| 43-70 | 0.94 (0.93, 0.95) | 0.24 (0.21, 0.27) | 0.06 (0.04, 0.11) | 0.17 (0.15, 0.20) | 0.15 (0.11, 0.19) | 0.11 (0.08, 0.17) | 0.77 (0.73, 0.82) |
| 71-98 | 1.32 (1.30, 1.34) | 0.32 (0.28, 0.36) | 0.07 (0.04, 0.12) | 0.16 (0.14, 0.19) | 0.16 (0.12, 0.22) | 0.09 (0.05, 0.14) | 0.82 (0.77, 0.88) |
| 99-126 | 1.75 (1.71, 1.79) | 0.43 (0.38, 0.49) | 0.10 (0.05, 0.19) | 0.22 (0.19, 0.26) | 0.25 (0.19, 0.33) | 0.22 (0.15, 0.33) | 0.80 (0.75, 0.85) |
| 127-154 | 1.96 (1.88, 2.05) | 0.48 (0.42, 0.56) | 0.09 (0.04, 0.19) | 0.25 (0.21, 0.30) | 0.34 (0.24, 0.47) | 0.26 (0.17, 0.39) | 0.77 (0.72, 0.82) |
| 155-182 | 1.91 (1.77, 2.05) | 0.56 (0.48, 0.65) | 0.18 (0.10, 0.32) | 0.35 (0.29, 0.41) | 0.46 (0.34, 0.63) | 0.25 (0.16, 0.39) | 0.83 (0.78, 0.89) |


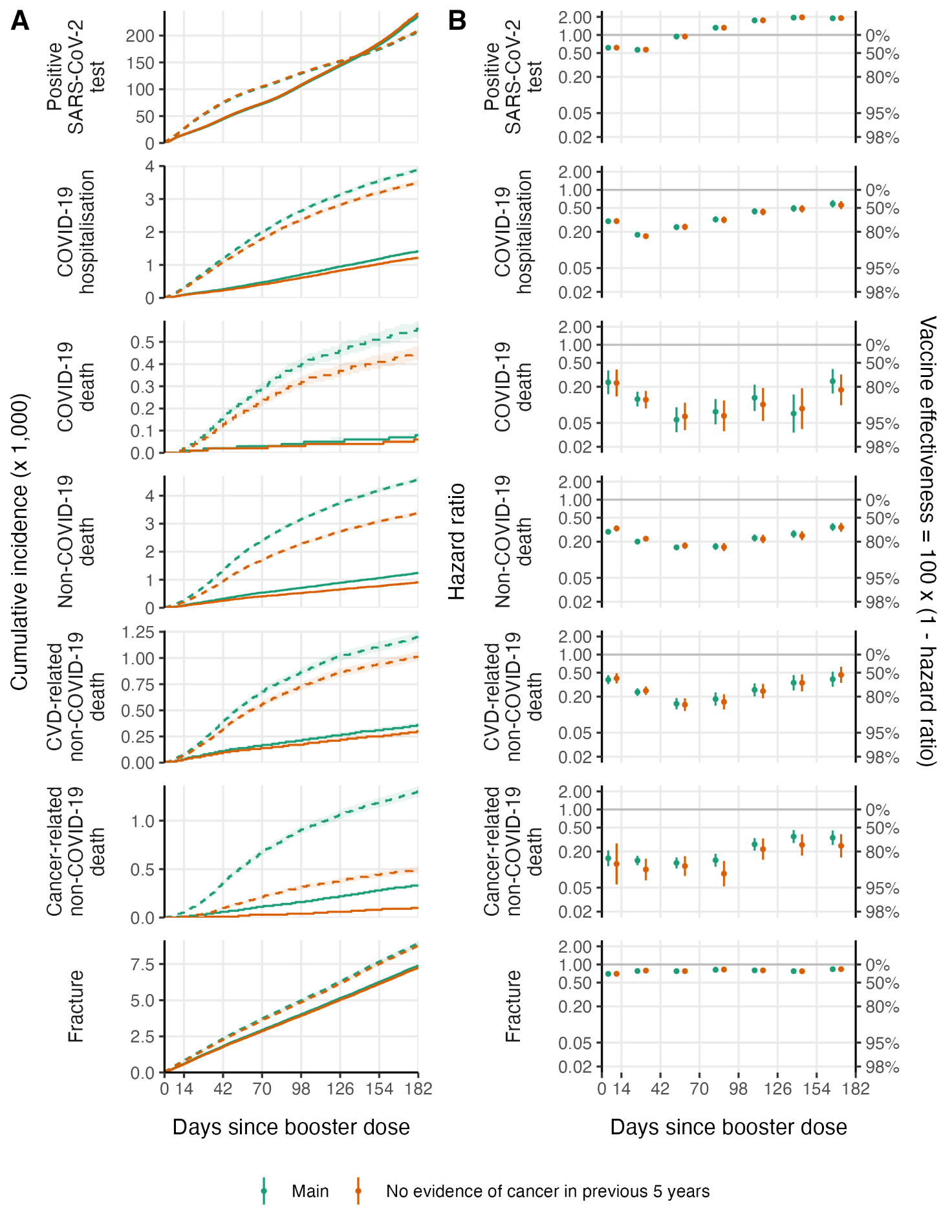


eFigure 6. Comparison of estimates from main cohort and non-cancer subgroup: (A) Kaplan-Meier estimates of cumulative incidence in matched boosted and unboosted treatment groups, without further adjustment for confounders. Dashed line = unboosted, solid line = boosted. (B) Cox hazard ratio estimates for boosted vs unboosted, with adjustment for confounders.

### References

1. NHS Digital. Data Security and Protection Toolkit. NHS Digital. Accessed April 30, 2020. https://digital.nhs.uk/data-and-information/looking-after-information/data-security-and-information-governance/data-security-and-protection-toolkit

2. NHS Digital. ISB1523: Anonymisation Standard for Publishing Health and Social Care Data. NHS Digital. Accessed April 30, 2020. https://digital.nhs.uk/data-and-information/information-standards/information-standards-and-data-collections-including-extractions/publications-and-notifications/standards-and-collections/isb1523-anonymisation-standard-for-publishing-health-and-social-care-data

3. Secretary of State for Health and Social Care. Coronavirus (COVID-19): notification to organisations to share information. UK Government. https://web.archive.org/web/20200421171727/https://www.gov.uk/government/publications/coronavirus-covid-19-notification-of-data-controllers-to-share-information

4. Secretary of State for Health and Social Care-UK Government. Coronavirus (COVID-19): notification to organisations to share information. Gov.uk. Published October 18, 2022. Accessed November 29, 2022. https://www.gov.uk/government/publications/coronavirus-covid-19-notification-to-organisations-to-share-information/coronavirus-covid-19-notice-under-regulation-34-of-the-health-service-control-of-patient-information-regulations-2002

5. Health Research Authority. Confidentiality Advisory Group. NHS Health Research Authority. Accessed November 29, 2022. https://www.hra.nhs.uk/about-us/committees-and-services/confidentiality-advisory-group/
